# Chemical Synthesis and Structure-Activity Relationship Study Yield Desotamide a Analogues with Improved Antibacterial Activity

**DOI:** 10.3390/md19060303

**Published:** 2021-05-24

**Authors:** Run Xu, Yongxiang Song, Jun Li, Jianhua Ju, Qinglian Li

**Affiliations:** 1 CAS Key Laboratory of Tropical Marine Bio-Resources and Ecology, Guangdong Key Laboratory of Marine Materia Medica, RNAM Center for Marine Microbiology, South China Sea Institute of Oceanology, Chinese Academy of Sciences, 164 West Xingang Road, Guangzhou 510301, China; runxu126@126.com (R.X.); songx@scsio.ac.cn (Y.S.); lijun@scsio.ac.cn (J.L.); jju@scsio.ac.cn (J.J.); 2 School of Pharmacy, Zunyi Medical University, 201 Dalian Road, Zunyi 563000, China; 3 College of Oceanology, University of Chinese Academy of Sciences, 19 Yuquan Road, Beijing 100049, China

**Keywords:** drug-resistant bacteria, desotamides, cyclohexapeptides, antibacterial, solid-phase peptide synthesis, structure-activity relationship

## Abstract

Desotamides A, a cyclohexapeptide produced by the deep-sea-derived *Streptomyces scopuliridis* SCSIO ZJ46, displays notable antibacterial activities against strains of *Streptococcus pnuemoniae*, *Staphylococcus aureus*, and methicillin-resistant *Staphylococcus epidermidis* (MRSE). In this study, to further explore its antibacterial potential and reveal the antibacterial structure-activity relationship of desotamides, 13 cyclopeptides including 10 new synthetic desotamide A analogues and wollamides B/B1/B2 were synthesized and evaluated for their antibacterial activities against a panel of Gram-positive and -negative pathogens. The bioactivity data reveal that residues at position II and VI greatly impact antibacterial activity. The most potent antibacterial analogues are desotamide A4 (**13**) and A6 (**15**) where l-*allo*-Ile at position II was substituted with l-Ile and Gly at position VI was simultaneously replaced by d-Lys or d-Arg; desotamides A4 (**13**) and A6 (**15**) showed a 2–4-fold increase of antibacterial activities against a series of Gram-positive pathogens including the prevalent clinical drug-resistant pathogen methicillin-resistant *Staphylococcus aureus* (MRSA) with MIC values of 8–32 μg/mL compared to the original desotamide A. The enhanced antibacterial activity, broad antibacterial spectrum of desotamides A4 and A6 highlighted their potential as new antibiotic leads for further development.

## 1. Introduction

Bacterial infection has always been an urgent threat to human health care, and this threat is worsening by the rapid emergence and spread of antibiotic-resistant bacteria such as methicillin-resistant *Staphylococcus aureus* (MRSA), vancomycin-resistant *Enterococcus* (VRE) and penicillin-resistant *Streptococcus*, as well as the multidrug-resistant Gram-negative bacteria [1,2,3]. In 2016, a Review on Antimicrobial Resistance reported that at least 0.7 million people lost their lives each year because of antibiotic resistance, and even 10 million people will die if there is no any action to control this situation [4]. Paradoxically, despite a growing clinic need, the discovery of antibiotics for treatments of bacterial infections slowed dramatically, with a limited number of new scaffolds marketed in the past forty years [5]. There is therefore an urgent need to continuously search for new classes of antibiotics with novel mechanisms of action to combat the growing antibiotic resistance.

Natural products of microbial origin are privileged in the sphere of antibiotic development and underpin the majority of antibiotics in clinical use [6,7,8]. Among them, the naturally occurring cyclopeptides play a pivotal role and attract significant attention due to their intriguing chemical structure and promising biological activities, such as tyrocidine-A [9], daptomycin [10], vancomycin [11]. The cyclic hexapeptides desotamides represent one of such class of cyclopeptides. Desotamides A–D are produced by the deep-sea-derived *Streptomyces scopuliridis* SCSIO ZJ46, of which desotamides A (**1**) and B (**2**) display notable antibacterial activities against strains of *Streptococcus pnuemoniae*, *Staphylococcus aureus*, and methicillin-resistant *Staphylococcus epidermidis* (MRSE) [12]. Two additional desotamide analogues [desotamides E (**3**) and F (**4**)] and related wollamides A (**6**) and B (**7**) were discovered from an Australia soil *Streptomyces* nov. sp. (MST-115088) [13]. These two desotamides as well as the wollamides were reported to display antibacterial activities against *Bacillus subtilis* and/or *Staphylococcus aureus*. A further desotamide analogue, desotamide G (**5**), was generated by heterologous expression of desotamide biosynthetic gene cluster in *Streptomyces coelicolor* M1152 [14]. Notably, the destomides and wollamides are noncytotoxic to mammalian cell lines (IC_50_ > 30 μM) [12,13], indicating selectivity for bacteria. Moreover, the antimicrobial target for these cyclic peptides remains unknown [15]. Therefore, desotamides represent excellent candidates for development as an antibacterial drug with potential human applications.

In this paper, to further investigate the structure-activity relationship (SAR) and explore the potential of desotamide A (**1**) as candidates of antibacterial drug development, we report the synthesis of 10 new desotamide A analogues (**10**–**19**) and their antibacterial properties.

## 2. Results and Discussion

### 2.1. Analogue Design

Comparison of the naturally produced desotamides, a preliminary SAR of this class of compounds has been defined. Desotamides A and B displayed antibacterial activities that were not seen with desotamides C and D [12], revealing that the Trp moiety at position V is essential for antibacterial activity. The comparably antibacterial activities of desotamide A and B suggested that substitute of lipophilic and bulky aliphatic group at position II was tolerated. In addition, unlike desotamide A, desotamide G display no notable antibacterial activities (MIC > 113 μg/mL) [12,14], suggesting that amino acid substitution at the position I is less tolerant. Furthermore, wollamides, structurally related to desotamides but possess the basic amino acid ornithine at position VI, were found to exhibit activity against mycobacteria that were not seen with desotamides [13], highlighted the importance of this amino acid for activity at position VI.

**Figure 1:**
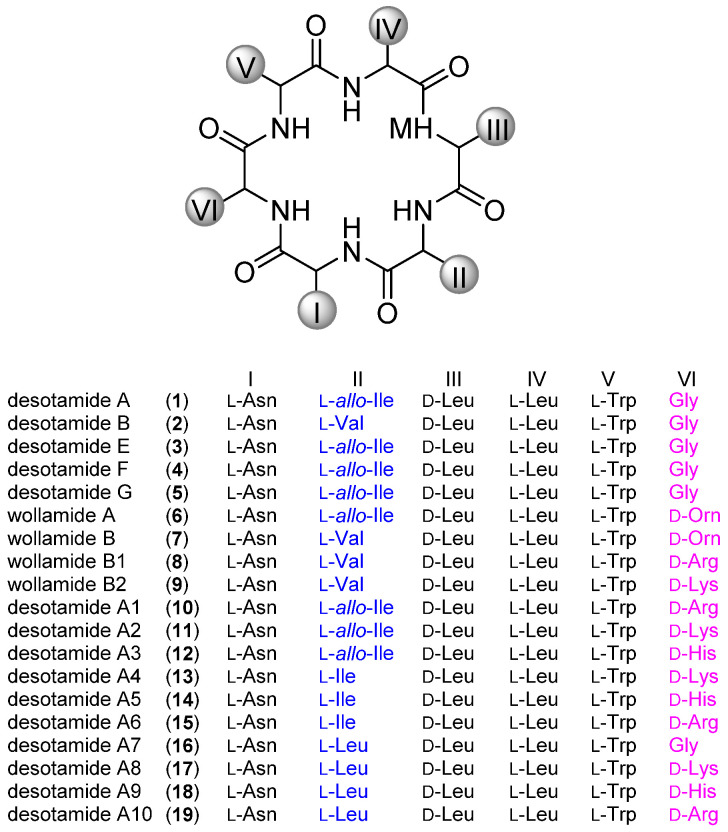
Chemical structures for natural products desotamides (**1–5**), wolloamides (**6–7**) and their synthesized analogues (**8–19**).

Based on these concerns, we selected desotamide A within these scaffolds as the most promising lead for further analogue design. Three series of compounds were designed based on this scaffold (Figure 1).

Series i: Analogues were designed and synthesized by substituting Gly at position VI with the basic amino acids d-Arg, d-Lys and d-His, respectively, generating the corresponding desotamides A1–A3 (**10**–**12**).

Series ii: Analogues were designed and synthesized by replacing the amino acids in both position II and position VI. In position II, the l-*allo*-Ile was replaced with the l-Ile; at the same time, Gly at position VI was substituted for the basic amino acids d-Arg, d-Lys and d-His, respectively, thereby generating desotamides A4–A6 (**13**–**15**).

Series iii: Analogues in this series included a single amino acid change at position II or double amino acid changes at both positions II and VI. Desotamide A7 (**16**) was generated by replacing l-*allo*-Ile at position II to l-Leu; desotamides A8–A10 (**17**–**19**) were synthesized by substituting l-*allo*-Ile at position II for l-Leu as well as Gly at position VI to the basic amino acids d-Arg, d-Lys and d-His, respectively.

In addition to the above 10 desotamide analogues, wollamides B, B1 and B2 [16] were also synthesized. Desotamide A was isolated from *S. scopuliridis* SCSIO ZJ46 as described previously [12] as control.

### 2.2. Synthesis

Synthesis of all the target cyclopeptides were achieved in three steps: (i) on-resin construction of the linear hexapeptide precursor; (ii) cleavage of linear precursor from the resin and deprotection of all the reactive amino acid side chains; (iii) macrocyclization of the purified linear precursor in solution phase. Scheme 1 outlines the representative synthesis of desomtmide A1 (**10**). The synthesized desomtmide A1 (**10**) was confirmed by HRESIMS, ^1^H and ^13^C NMR analyses (Figures S1–S39). Using this standard synthetic route, all the other cyclohexapeptides (**7**–**9** and **11**–**19**) were synthesized.

### 2.3. Antibacterial Activity

**Figure 2:**
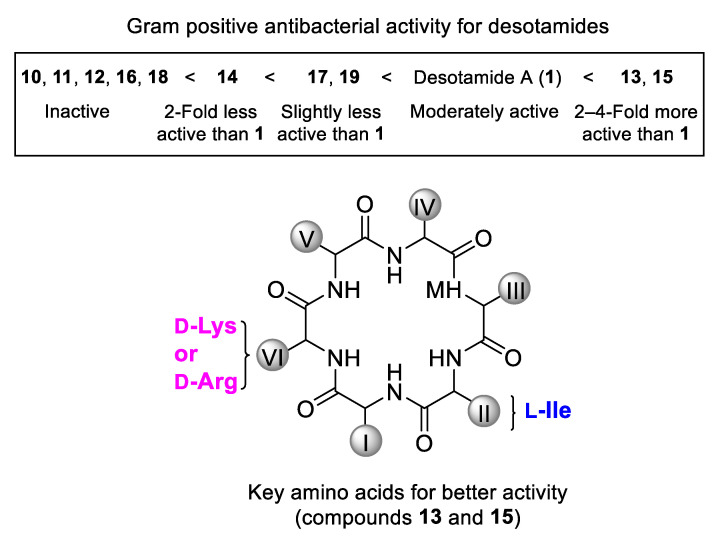
Summary of antibacterial properties and key amino acids for an improved activity for desotamide analogues.

**Figure 3:**
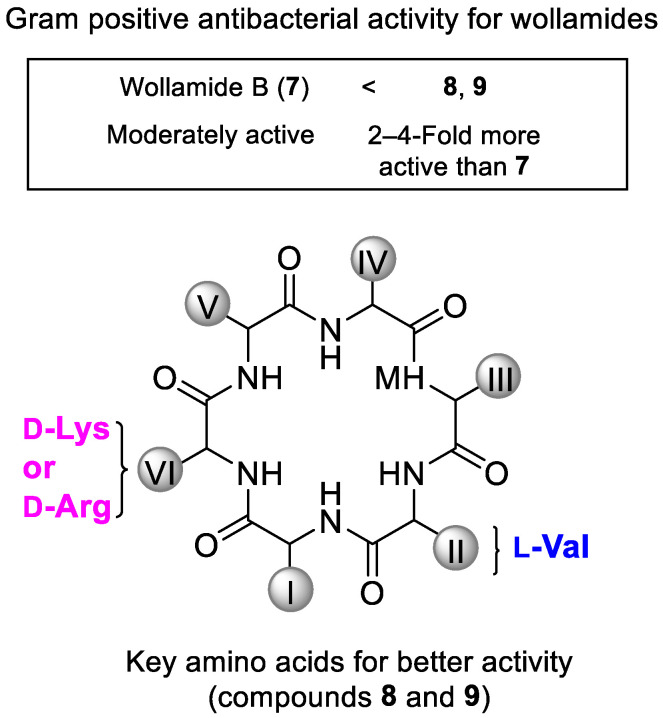
Summary of antibacterial properties and key amino acids for an improved activity for wollamide analogues.

All the synthesized cyclopeptides (**7**–**19**) and the desotamide A (**1**) isolated from *S. scopuliridis* SCSIO ZJ46 were evaluated for antibacterial activities against a series of Gram-positive pathogens including 5 clinical pathogens *Staphylococcus aureus* ATCC29213, *Bacillus subtilis* BS01, *Micrococcus luteus* ML01, *Enterococcus faecalis* ATCC29212; 3 drug-resistant poultry pathogens *Staphylococcus simulans* LJH13, *Enterococcus gallinarum* LJH11, *Staphylococcus haemolyticus* LJH9; 4 clinical methicillin-resistant *Staphylococcus aureus* (MRSA) shhs-E1, MRSA 16339, MRSA 745524, MRSA 16162. Their antibacterial activities against the Gram-positive pathogens *Escherichia coli* ATCC 13,124 and clinical drug-resistant *Klebsiella pneumonia* 15580 were also assayed. The antibacterial activities were summarised in Table 1, Figure 2 and Figure 3.

The bioactivity data showed that deostamide A (**1**) displayed moderately antibacterial activities against Gram-positive bacteria. The desotamide A analogues (**10–12**) with Gly at position VI substituted with the basic amino acids d-Arg, d-Lys and d-His, respectively, were inactive again all the Gram-positive bacteria tested. This result is consistent with the previous report that wollamide A with l-*allo*-Ile at position II and the basic amino acid d-Orn at position VI displayed no antibacterial against *Staphylococcus aureus* [13], suggesting that the basic amino acids at position VI were not tolerated when l-*allo*-Ile is present at position II. However, when the l-*allo*-Ile at position II was replaced by l-Ile, the simultaneous substitution at position VI with basic amino acids d-Lys (**13**) or d-Arg (**15**), resulted in a 2–4-fold increase of activities against the entire test panel of Gram-positive bacteria. Although d-His also belongs to the basic amino acid just as d-Lys or d-Arg, the substitution of Gly with d-His (**14**) generally showed 2-fold less potent MIC value compared to **1**. This finding indicates that residues at position II and VI greatly impacted antibacterial activity. To further validate the importance of l-Ile at position II and d-Lys/d-Arg at position VI, l-*allo*-Ile was substituted with another aliphatic amino acid l-Leu (**16**), and Gly was also simultaneously exchanged by basic amino acids d-Lys (**17**), d-His (**18**) and d-Arg (**19**), respectively. Compounds **17** and **19** retained the antibacterial activity slightly less potent than **1**, whereas compounds **16** and **18** completely loss of activity with MIC value > 128 μg/mL (Figure 2). With respect to the anti-Gram-negative activity, all the desotamide analogues (**10**–**19**), as well as the original desotamide A (**1**), were generally inactive against the Gram-negative pathogens tested, with only compounds **13** and **15**, which are the most potent analogues against Gram-positive bacteria, having a weak activity (MIC = 64–128μg/mL). Taken together, these results strongly supported the II-l-Ile along with VI-d-Lys/d-Arg substitutions were favourable for the antibacterial activity.

Wollamide B (**7**) and its two synthesized analogues, wollamides B1 (**8**) and B2 (**9**) showed a similar anti-Gram-positive profile as desotamide analogues described above. Wollamide B (**7**) with l-Val at position II and the basic amino acid d-Orn at position VI displayed comparably antibacterial activity to desotamide A (**1**). When the VI-d-Orn was replaced by d-Arg (**8**) or d-Lys (**9**), respectively, the anti-Gram-positive activity increases 2–4 folds compared to wollamide B (**7**) (Table 1 and Figure 3). This finding is consistent with the previous report that wollamide B1 (**8**) and B2 (**9**) show more potent against mycobacteria than wollamide B (**7**) [16] as well as our observation that desotamides A4 (**13**) and A6 (**15**) display more potent against Gram-positive bacteria, indicating the importance of basic amino acid d-Lys/d-Arg for the antibacterial activity within both desotamide and wollamide family of antibiotics.

## 3. Materials and Methods

### 3.1. General Experimental Procedures

All the chemicals and reagents used for synthesis were purchased from a commercial source. Monitoring of the reaction progress in solution-phase and final purity of synthetic compounds were detected by analytic HPLC using a Kromasil ODS column (250 × 4.6 mm, 5 μm) eluted with a gradient of 0–100% solvent B (solvent B: CH_3_CN; solvent A: ddH_2_O) at a flow rate 1 mL/min with UV detection at 220 nm. The synthetic compounds were purified by preparative RP-HPLC using an sp-120-10-ODS-RPS column (250 × 100 mm, 10 μm). High-resolution mass spectra were obtained on a Maxis quadrupole-time-of-flight mass spectrometer (Bruker). NMR spectra were recorded on Avance 500 MHz spectrometer instrument (21 May 2021, Bruker, Zurich, Switzerland).

### 3.2. General Procedure for the Solid-Phase Synthesis of Cyclic Hexapeptides

The first Fmoc-protected amino acid (1.5 equiv.), HBTU (3 equiv.) and DIEA (3 equiv.) were added into a solution of 2-chlorotrityl chloride (2-CTC) in DMF at room temperature. After reaction for 1.5 h, the mixture was capped with methanol for 0.5 h. The reaction was washed three times with DCM and DMF (40 mL, 1 min each), then concentrated under vacuum, and redissolved with 20% piperidine in DMF for the removal of the first Fmoc group. Subsequently, elongation of the peptides was achieved with another Fmoc-amino acid using a mixture of HBTU and DIEA in DMF. After 0.5 h, the mixture was washed three times with DMF and then DCM. The procedure of deprotection, washing, coupling and washing was repeated for the synthesis of the linear hexapeptide. The cleavage of linear precursor from the resin and final global deprotection were processed with a solution of TFA/thioanisole/phenol/ddH_2_O (33/2/2/1/2, *v/v*). After stirring in dark for 2 h, the reaction mixture was filtrated and subsequently washed with TFA. The addition of pre-chilled diethyl ether and centrifugation afforded the crude precipitated product, which was then washed three times with pre-chilled diethyl ether. The crude linear hexapeptide was purified with RP-HPLC (0.1% TFA of CH_3_CN/H_2_O system) at a flow rate of 23 mL/min. For macrocyclization, the purified linear hexapeptides were first dissolved in DMF to give a 10^−3^–10^−4^ M solution and HBTU was added to this solution under stirring. DIEA was then added to this dilute solution to adjust the pH value to 8.0–9.0. The reaction mixture was stirred at room temperature for 2 d. Following these, the reaction mixture was concentrated under vacuum before purification by RP-HPLC.

Wollamide B (**7**): cyclo(Asn-Val-d-Leu-Leu-Trp-d-Orn). ^1^H NMR (DMSO-*d6*, 400 MHz) *δ* 10.85 (br d, 1H, *J* = 1.6 Hz), 8.38 (br d, 1H, *J* = 5.6 Hz), 8.35 (br d, 1H, *J* = 7.9 Hz), 8.29 (br d, 1H, *J* = 7.9 Hz), 7.69 (br d, 1H, *J* = 7.5 Hz), 7.6–7.7 (m, 2H), 7.56 (br s, 1H), 7.53 (d, 1H, *J* = 7.8 Hz), 7.49 (br d, 1H, *J* = 8.2 Hz), 7.39 (br d, 1H, *J* = 8.2 Hz), 7.33 (d, 1H, *J* = 8.0 Hz), 7.14 (d, 1H, *J* = 2.1 Hz), 7.0–7.1 (m, 1H), 7.0–7.0 (m, 1H), 7.0–7.0 (m, 1H), 4.6–4.6 (m, 1H), 4.44 (q, 1H, *J* = 7.1 Hz), 4.3–4.4 (m, 2H), 4.1–4.2 (m, 1H), 4.02 (dd, 1H, *J* = 4.3, 7.6 Hz), 3.19 (br dd, 1H, *J* = 4.1, 14.6 Hz), 2.96 (br dd, 1H, *J* = 10.3, 14.6 Hz), 2.6–2.7 (m, 4H), 2.2–2.3 (m, 1H), 1.4–1.6 (m, 8H), 1.2–1.4 (m, 2H), 0.8–0.9 (m, 18H). ^13^C NMR (DMSO-*d6*, 101 MHz) *δ* 173.5, 171.8, 171.6, 171.2, 170.7, 170.6, 170.6, 136.1, 127.0, 123.6, 120.9, 118.4, 118.1, 111.4, 110.0, 58.6, 55.2, 51.9, 51.8, 50.4, 49.5, 41.9, 40.4, 38.3, 37.1, 28.8, 27.2, 26.6, 24.4, 24.1, 23.2, 22.6, 22.5, 22.4, 22.1, 19.1, 17.0. (+)-HRESIMS *m/z* 740.4445 [M + H]^+^ (calcd for C_37_H_58_N_9_O_7_, 740.4454).

Wollamide B1 (**8**): cyclo(Asn-Val-d-Leu-Leu-Trp-d-Arg). ^1^H NMR (DMSO-*d_6_*, 400 MHz) *δ* 10.80 (s, 1H), 8.38 (br d, 1H, *J =* 5.4 Hz), 8.35 (br d, 1H, *J =* 7.7 Hz), 8.30 (br d, 1H, *J =* 8.0 Hz), 7.72 (br d, 1H, *J =* 7.2 Hz), 7.54 (br s, 1H), 7.52 (br d, 1H, *J =* 8.0 Hz), 7.4–7.5 (m, 4H), 7.33 (d, 1H, *J =* 8.0 Hz), 7.14 (br d, 1H, *J =* 1.8 Hz), 7.06 (br t, 2H, *J =* 7.3 Hz), 7.0–7.0 (m, 3H), 4.5–4.7 (m, 1H), 4.45 (q, 1H, *J =* 7.2 Hz), 4.3–4.3 (m, 2H), 4.12 (q, 1H, *J =* 7.2 Hz), 4.00 (dd, 1H, *J =* 4.3, 7.5 Hz), 3.21 (br dd, 1H, *J =* 3.8, 14.6 Hz), 3.02 (dt, 1H, *J =* 4.0, 6.5 Hz), 2.9–3.0 (m, 4H), 2.2–2.3 (m, 1H), 1.4–1.6 (m, 9H), 1.0–1.2 (m, 1H), 0.8–0.9 (m, 18H).^13^C NMR (DMSO-*d_6_*, 101 MHz) *δ* 173.5, 171.7, 171.6, 171.3, 170.7, 170.6, 170.6, 156.7, 136.1, 127.0, 123.7, 120.9, 118.4, 118.1, 111.3, 110.0, 58.6, 55.1, 52.3, 51.9, 50.5, 49.5, 42.0, 40.4, 40.2, 37.2, 28.7, 27.2, 26.9, 24.5, 24.4, 24.1, 22.6, 22.5, 22.4, 22.1, 19.1, 17.0. (+)-HRESIMS *m/z* 782.4674 [M + H]^+^ (calcd for C_38_H_60_N_11_O_7_, 782.4672).

Wollamide B2 (**9**): cyclo(Asn-Val-d-Leu-Leu-Trp-d-Lys). ^1^H NMR (DMSO-*d_6_*, 400 MHz) *δ* 10.84 (d, 1H, *J =* 1.8 Hz), 8.3–8.4 (m, 2H), 8.28 (br d, 1H, *J =* 8.0 Hz), 7.67 (br s, 2H), 7.65 (br s, 1H), 7.53 (br d, 2H, *J =* 7.2 Hz), 7.45 (br d, 1H, *J =* 8.0 Hz), 7.4–7.4 (m, 1H), 7.33 (d, 1H, *J =* 8.0 Hz), 7.15 (d, 1H, *J =* 2.0 Hz), 7.06 (t, 1H, *J =* 7.7 Hz), 7.00 (s, 1H), 6.98 (t, 1H, *J =* 7.7 Hz), 4.6–4.6 (m, 1H), 4.45 (q, 1H, *J =* 7.1 Hz), 4.2–4.4 (m, 2H), 4.09 (q, 1H, *J =* 7.2 Hz), 3.99 (dd, 1H, *J =* 4.4, 7.5 Hz), 3.22 (br dd, 1H, *J =* 3.8, 14.5 Hz), 2.93 (br dd, 1H, *J =* 10.7, 14.4 Hz), 2.5–2.7 (m, 4H), 2.2–2.3 (m, 1H), 1.3–1.7 (m, 11H), 1.0–1.1 (m, 1H), 0.8–0.9 (m, 18H). ^13^C NMR (DMSO-*d_6_*, 101 MHz) *δ* 173.5, 171.7, 171.6, 171.4, 170.8, 170.6, 170.5, 136.1, 127.0, 123.7, 120.9, 118.3, 118.1, 111.3, 110.1, 58.6, 55.1, 52.3, 51.9, 50.5, 49.5, 42.0, 38.6, 38.6, 37.2, 29.2, 28.8, 27.1, 26.4, 24.5, 24.1, 22.6, 22.5, 22.5, 22.1, 21.7, 19.1, 17.0. (+)-HRESIMS *m/z* 754.4609 [M + H]^+^ (calcd for C_38_H_60_N_9_O_7_, 754.4610).

Desotamide A1 (**10**): cyclo(Asn-*allo*-Ile-d-Leu-Leu-Trp-d-Arg). ^1^H NMR (DMSO-*d_6_*, 400 MHz) *δ* 10.8–10.8 (m, 1H), 8.29 (br d, 1H, *J =* 6.9 Hz), 8.20 (br d, 1H, *J =* 8.3 Hz), 7.98 (br d, 1H, *J =* 7.4 Hz), 7.88 (br d, 1H, *J =* 7.4 Hz), 7.57 (d, 1H, *J =* 7.8 Hz), 7.52 (br d, 1H, *J =* 7.3 Hz), 7.4–7.5 (m, 4H), 7.33 (d, 1H, *J =* 8.0 Hz), 7.13 (d, 1H, *J =* 2.1 Hz), 7.0–7.1 (m, 2H), 6.9–7.0 (m, 3H), 4.48 (q, 1H, *J =* 6.8 Hz), 4.3–4.4 (m, 1H), 4.3–4.3 (m, 1H), 4.19 (dd, 1H, *J =* 5.6, 7.9 Hz), 4.0–4.1 (m, 2H), 3.16 (br dd, 1H, *J =* 5.0, 14.5 Hz), 3.0–3.1 (m, 1H), 2.95 (br d, 2H, *J =* 5.9 Hz), 1.86 (td, 1H, *J =* 6.4, 13.1 Hz), 1.2–1.7 (m, 12H), 1.1–1.2 (m, 1H), 1.0–1.1 (m, 1H), 0.89 (br t, 6H, *J =* 6.7 Hz), 0.8–0.9 (m, 5H), 0.8–0.8 (m, 4H), 0.77 (d, 3H, *J =* 6.9 Hz). ^13^C NMR (DMSO-*d_6_*, 101 MHz) *δ* 172.1, 171.9, 171.4, 171.0, 170.9, 170.6, 170.5, 156.6, 136.1, 127.1, 123.6, 120.9, 118.3, 118.3, 111.3, 110.2, 55.7, 55.0, 52.8, 52.5, 51.4, 49.7, 40.9, 40.4, 40.4, 36.5, 36.3, 27.7, 27.4, 25.6, 24.6, 24.3, 24.2, 22.9, 22.6, 22.5, 21.0, 14.8, 11.5. (+)-HRESIMS *m/z* 796.4832 [M + H]^+^ (calcd for C_39_H_62_N_11_O_7_, 796.4828).

Desotamide A2 (**11**): cyclo(Asn-*allo*-Ile-d-Leu-Leu-Trp-d-Lys). ^1^H NMR (DMSO-*d_6_*, 400 MHz) *δ* 10.8–10.9 (m, 1H), 8.2–8.3 (m, 1H), 8.19 (br d, 1H, *J =* 8.3 Hz), 8.00 (br d, 1H, *J =* 7.3 Hz), 7.83 (br d, 1H, *J =* 7.4 Hz), 7.67 (br s, 2H), 7.58 (d, 1H, *J =* 7.8 Hz), 7.51 (br d, 1H, *J =* 7.3 Hz), 7.4–7.5 (m, 1H), 7.37 (br d, 1H, *J =* 8.2 Hz), 7.3–7.3 (m, 1H), 7.14 (d, 1H, *J =* 2.0 Hz), 7.0–7.1 (m, 1H), 7.0–7.0 (m, 1H), 6.9–7.0 (m, 1H), 4.47 (q, 1H, *J =* 6.9 Hz), 4.3–4.4 (m, 1H), 4.3–4.3 (m, 1H), 4.18 (dd, 1H, *J =* 5.8, 8.0 Hz), 4.1–4.1 (m, 1H), 4.0–4.1 (m, 1H), 3.16 (br dd, 1H, *J =* 4.9, 14.6 Hz), 2.99 (br dd, 1H, *J =* 9.9, 14.5 Hz), 2.4–2.7 (m, 4H), 1.85 (td, 1H, *J =* 6.4, 13.1 Hz), 1.6–1.7 (m, 1H), 1.4–1.6 (m, 6H), 1.2–1.4 (m, 5H), 1.0–1.1 (m, 2H), 0.9–0.9 (m, 3H), 0.88 (br s, 1H), 0.8–0.9 (m, 3H), 0.8–0.8 (m, 1H), 0.8–0.8 (m, 3H), 0.77 (d, 3H, *J =* 6.8 Hz).^13^C NMR (DMSO-*d_6_*, 101 MHz) *δ* 172.1, 171.8, 171.4, 171.0, 170.8, 170.7, 170.4, 136.1, 127.2, 123.6, 120.8, 118.3, 118.2, 111.3, 110.2, 55.7, 55.1, 52.8, 52.6, 51.4, 49.7, 40.8, 40.4, 38.5, 36.5, 36.3, 30.0, 27.3, 26.5, 25.6, 24.2, 24.2, 22.9, 22.6, 22.5, 21.8, 21.0, 14.7, 11.5. (+)-HRESIMS *m/z* 768.4763 [M + H]^+^ (calcd for C_39_H_62_N_9_O_7_, 768.4767).

Desotamide A3 (**12**): cyclo(Asn-*allo*-Ile-d-Leu-Leu-Trp-d-His). ^1^H NMR (DMSO-*d_6_*, 400 MHz) *δ* 14.14 (br s, 2H), 10.84 (s, 1H), 8.9–8.9 (m, 1H), 8.26 (br d, 1H, *J =* 6.9 Hz), 8.19 (br d, 1H, *J =* 8.2 Hz), 8.07 (br dd, 2H, *J =* 3.5, 7.5 Hz), 7.63 (br d, 1H, *J =* 7.0 Hz), 7.5–7.6 (m, 3H), 7.33 (d, 1H, *J =* 8.2 Hz), 7.13 (s, 1H), 7.1–7.1 (m, 1H), 7.0–7.1 (m, 1H), 6.9–7.0 (m, 2H), 4.4–4.5 (m, 2H), 4.3–4.4 (m, 1H), 4.25 (q, 1H, *J =* 6.9 Hz), 4.1–4.2 (m, 1H), 4.1–4.1 (m, 1H), 3.1–3.2 (m, 1H), 2.9–3.1 (m, 2H), 2.76 (br dd, 1H, *J =* 9.0, 15.2 Hz), 1.85 (td, 1H, *J =* 6.5, 12.9 Hz), 1.6–1.7 (m, 1H), 1.4–1.6 (m, 6H), 1.3–1.4 (m, 1H), 1.0–1.2 (m, 2H), 0.89 (br d, 3H, *J =* 6.8 Hz), 0.87 (br d, 3H, *J =* 6.3 Hz), 0.8–0.9 (m, 9H), 0.78 (d, 3H, *J =* 6.8 Hz). ^13^C NMR (DMSO-*d_6_*, 101 MHz) *δ* 172.3, 171.9, 171.6, 171.0, 170.7, 170.6, 169.2, 136.1, 133.6, 129.3, 127.1, 123.5, 120.9, 118.3, 116.6, 111.3, 109.9, 56.0, 55.2, 52.6, 51.6, 51.5, 49.6, 40.7, 40.4, 40.4, 36.4, 27.5, 25.8, 25.5, 24.2, 22.9, 22.5, 22.4, 21.0, 14.9, 11.5. (+)-HRESIMS *m/z* 777.4403 [M + H]^+^ (calcd for C_39_H_57_N_10_O_7_, 777.4406).

Desotamide A4 (**13**): cyclo(Asn-Ile-d-Leu-Leu-Trp-d-Lys). ^1^H NMR (DMSO-*d_6_*, 400 MHz) *δ* 10.84 (s, 1H), 8.3–8.4 (m, 2H), 8.25 (br d, 1H, *J =* 7.9 Hz), 7.66 (br s, 2H), 7.63 (br d, 1H, *J =* 7.3 Hz), 7.55 (br s, 1H), 7.5–7.5 (m, 1H), 7.4–7.5 (m, 1H), 7.4–7.4 (m, 1H), 7.33 (d, 1H, *J =* 8.2 Hz), 7.15 (d, 1H, *J =* 2.0 Hz), 7.0–7.1 (m, 1H), 7.0–7.0 (m, 1H), 6.9–7.0 (m, 1H), 4.5–4.6 (m, 1H), 4.43 (q, 1H, *J =* 7.1 Hz), 4.2–4.3 (m, 2H), 4.0–4.1 (m, 1H), 4.01 (dd, 1H, *J =* 4.5, 7.2 Hz), 3.20 (br dd, 1H, *J =* 3.9, 14.6 Hz), 2.93 (br dd, 1H, *J =* 10.6, 14.5 Hz), 2.6–2.7 (m, 4H), 1.9–2.0 (m, 1H), 1.2–1.6 (m, 13H), 1.0–1.1 (m, 1H), 0.8–0.9 (m, 18H);^13^C NMR (DMSO-*d_6_*, 101 MHz) *δ* 173.5, 171.7, 171.6, 171.4, 170.8, 170.5, 170.5, 136.1, 127.0, 123.7, 120.9, 118.3, 118.1, 111.3, 110.1, 58.3, 55.2, 52.3, 51.8, 50.5, 49.5, 41.8, 40.4, 38.6, 37.0, 35.5, 29.1, 27.1, 26.4, 24.5, 24.1, 23.9, 22.5, 22.5, 22.0, 21.7, 15.6, 11.8. (+)-HRESIMS *m/z* 768.4774 [M + H]^+^ (calcd for C_39_H_62_N_9_O_7_, 768.4767).

Desotamide A5 (**14**): cyclo(Asn-Ile-d-Leu-Leu-Trp-d-His). ^1^H NMR (DMSO-*d_6_*, 400 MHz) *δ* 14.0–14.2 (m, 1H), 10.8–10.9 (m, 1H), 8.9–8.9 (m, 1H), 8.4–8.5 (m, 1H), 8.44 (br s, 1H), 8.15 (br d, 1H, *J =* 8.2 Hz), 7.71 (br s, 1H), 7.59 (br dd, 2H, *J =* 3.0, 8.3 Hz), 7.52 (s, 1H), 7.5–7.5 (m, 1H), 7.33 (d, 1H, *J =* 8.2 Hz), 7.17 (s, 1H), 7.1–7.2 (m, 1H), 7.10 (d, 1H, *J =* 2.1 Hz), 7.0–7.1 (m, 1H), 6.9–7.0 (m, 1H), 4.5–4.6 (m, 2H), 4.43 (q, 1H, *J =* 7.4 Hz), 4.36 (dt, 1H, *J =* 5.0, 9.0 Hz), 4.3–4.3 (m, 1H), 4.06 (br dd, 1H, *J =* 4.3, 6.8 Hz), 3.1–3.2 (m, 1H), 3.0–3.1 (m, 1H), 3.0–3.0 (m, 1H), 2.8–2.9 (m, 2H), 2.63 (dd, 1H, *J =* 5.0, 16.0 Hz), 1.9–2.0 (m, 1H), 1.2–1.6 (m, 8H), 0.8–0.9 (m, 18H). ^13^C NMR (DMSO-*d_6_*, 101 MHz) *δ* 174.1, 172.0, 171.8, 171.1, 170.9, 170.8, 169.7, 136.1, 133.5, 129.2, 126.9, 123.5, 121.0, 118.4, 118.1, 116.9, 111.4, 109.8, 58.7, 55.7, 52.1, 51.1, 50.4, 49.6, 41.8, 36.4, 35.4, 27.2, 25.0, 24.5, 24.1, 23.9, 22.6, 22.4, 22.4, 22.1, 15.6, 11.8. (+)-HRESIMS *m/z* 777.4404 [M + H]^+^ (calcd for C_39_H_57_N_10_O_7_, 777.4406).

Desotamide A6 (**15**): cyclo(Asn-Ile-d-Leu-Leu-Trp-d-Arg). ^1^H NMR (DMSO-*d_6_*, 400 MHz) *δ* 10.8–10.8 (m, 1H), 8.34 (br s, 1H), 8.33 (br s, 1H), 8.26 (br d, 1H, *J =* 7.9 Hz), 7.68 (br d, 1H, *J =* 7.2 Hz), 7.56 (br s, 1H), 7.51 (d, 1H, *J =* 7.8 Hz), 7.4–7.5 (m, 4H), 7.33 (d, 1H, *J =* 8.0 Hz), 7.14 (d, 1H, *J =* 2.1 Hz), 7.0–7.1 (m, 2H), 6.9–7.0 (m, 3H), 4.5–4.6 (m, 1H), 4.43 (q, 1H, *J =* 7.2 Hz), 4.3–4.4 (m, 2H), 4.12 (q, 1H, *J =* 7.2 Hz), 4.02 (dd, 1H, *J =* 4.4, 7.3 Hz), 3.20 (br dd, 1H, *J =* 4.0, 14.6 Hz), 2.9–3.0 (m, 3H), 2.6–2.7 (m, 2H), 1.98 (dt, 1H, *J =* 4.6, 10.3 Hz), 1.2–1.6 (m, 13H), 1.0–1.2 (m, 1H), 0.91 (d, 7H, *J =* 6.3 Hz), 0.8–0.9 (m, 13H).^13^C NMR (DMSO-*d_6_*, 101 MHz) *δ* 173.5, 171.7, 171.6, 171.3, 170.8, 170.6, 170.5, 156.7, 136.1, 126.9, 123.7, 120.9, 118.4, 118.1, 111.3, 110.0, 58.3, 55.2, 52.2, 51.8, 50.5, 49.5, 41.8, 40.4, 40.2, 37.0, 35.5, 27.1, 26.9, 24.6, 24.5, 24.1, 23.9, 22.5, 22.5, 22.5, 22.0, 15.7, 11.8. (+)-HRESIMS *m/z* 796.4823 [M + H]^+^ (calcd for C_39_H_62_N_11_O_7_, 796.4828).

Desotamide A7 (**16**): cyclo(Asn-Ile-d-Leu-Leu-Trp-Gly). ^1^H NMR (DMSO-*d_6_*, 400 MHz) *δ* 10.83 (s, 1H), 8.62 (br d, 1H, *J =* 7.0 Hz), 8.34 (br d, 1H, *J =* 5.9 Hz), 8.2–8.3 (m, 1H), 7.88 (br t, 1H, *J =* 5.4 Hz), 7.69 (br d, 1H, *J =* 8.0 Hz), 7.67 (br s, 1H), 7.63 (br d, 1H, *J =* 8.4 Hz), 7.51 (d, 1H, *J =* 7.8 Hz), 7.32 (d, 1H, *J =* 8.0 Hz), 7.1–7.2 (m, 1H), 7.1–7.1 (m, 1H), 7.06 (t, 1H, *J =* 7.3 Hz), 6.98 (t, 1H, *J =* 7.3 Hz), 4.52 (td, 1H, *J =* 5.3, 8.3 Hz), 4.3–4.4 (m, 2H), 4.19 (q, 1H, *J =* 7.2 Hz), 4.07 (ddd, 1H, *J =* 4.1, 6.7, 10.5 Hz), 3.87 (br dd, 1H, *J =* 6.3, 16.1 Hz), 3.31 (br dd, 1H, *J =* 4.6, 16.0 Hz), 3.1–3.2 (m, 1H), 2.98 (br dd, 1H, *J =* 10.0, 14.6 Hz), 2.84 (br dd, 1H, *J =* 5.3, 16.0 Hz), 2.63 (br dd, 1H, *J =* 5.4, 15.9 Hz), 1.4–1.7 (m, 9H), 0.8–0.9 (m, 18H). ^13^C NMR (DMSO-*d_6_*, 101 MHz) *δ* 172.8, 172.1, 171.9, 171.5, 170.9, 170.9, 168.9, 136.1, 127.0, 123.5, 120.9, 118.3, 118.1, 111.3, 110.0, 55.3, 51.9, 51.9, 50.8, 49.2, 43.3, 41.5, 40.4, 40.4, 36.7, 27.4, 24.5, 24.2, 24.2, 23.1, 22.6, 22.5, 22.4, 22.1, 20.8. (+)-HRESIMS *m/z* 697.4025 [M + H]^+^ (calcd for C_35_H_53_N_8_O_7_, 697.4032).

Desotamide A8 (**17**): cyclo(Asn-Ile-d-Leu-Leu-Trp-d-Lys). ^1^H NMR (DMSO-*d_6_*, 400 MHz) *δ* 10.8–10.9 (m, 1H), 8.70 (br d, 1H, *J =* 7.0 Hz), 8.42 (br d, 1H, *J =* 5.4 Hz), 8.25 (br d, 1H, *J =* 8.3 Hz), 7.67 (br s, 2H), 7.59 (br d, 1H, *J =* 7.0 Hz), 7.55 (br s, 1H), 7.53 (d, 1H, *J =* 7.8 Hz), 7.46 (br d, 2H, *J =* 8.2 Hz), 7.33 (d, 1H, *J =* 8.2 Hz), 7.14 (d, 1H, *J =* 2.1 Hz), 7.0–7.1 (m, 1H), 7.02 (br s, 1H), 7.0–7.0 (m, 1H), 4.5–4.6 (m, 1H), 4.4–4.5 (m, 1H), 4.30 (ddd, 1H, *J =* 4.3, 8.2, 10.4 Hz), 4.1–4.2 (m, 1H), 4.0–4.1 (m, 1H), 4.0–4.0 (m, 1H), 3.21 (br dd, 1H, *J =* 3.9, 14.4 Hz), 2.93 (br dd, 1H, *J =* 10.6, 14.5 Hz), 2.5–2.7 (m, 4H), 1.3–1.7 (m, 14H), 1.0–1.2 (m, 1H), 0.9–0.9 (m, 12H), 0.84 (d, 3H, *J =* 6.5 Hz), 0.80 (d, 3H, *J =* 6.3 Hz);^13^C NMR (DMSO-*d_6_*, 101 MHz) *δ* 173.0, 171.8, 171.8, 171.5, 171.2, 170.8, 170.5, 136.1, 127.0, 123.7, 120.9, 118.3, 118.1, 111.3, 110.1, 55.1, 52.3, 52.1, 51.9, 50.5, 49.4, 42.0, 40.4, 38.6, 38.6, 37.2, 29.1, 27.2, 26.4, 24.5, 24.2, 24.1, 23.1, 22.7, 22.5, 22.4, 22.0, 21.7, 20.6. (+)-HRESIMS *m/z* 768.4768 [M + H]^+^ (calcd for C_39_H_62_N_9_O_7_, 768.4767).

Desotamide A9 (**18**): cyclo(Asn-Ile-d-Leu-Leu-Trp-d-His). ^1^H NMR (DMSO-*d_6_*, 400 MHz) *δ* 14.11 (br s, 2H), 10.86 (s, 1H), 8.91 (d, 1H, *J =* 1.0 Hz), 8.83 (br d, 1H, *J =* 6.5 Hz), 8.54 (br d, 1H, *J =* 4.8 Hz), 8.13 (br d, 1H, *J =* 8.2 Hz), 7.75 (s, 1H), 7.62 (br d, 1H, *J =* 8.5 Hz), 7.55 (br dd, 2H, *J =* 3.8, 8.2 Hz), 7.51 (d, 1H, *J =* 7.8 Hz), 7.34 (d, 1H, *J =* 8.0 Hz), 7.18 (s, 1H), 7.16 (br s, 1H), 7.09 (s, 1H), 7.0–7.1 (m, 1H), 7.0–7.0 (m, 1H), 4.5–4.5 (m, 2H), 4.4–4.5 (m, 1H), 4.37 (dt, 1H, *J =* 5.0, 9.1 Hz), 4.1–4.2 (m, 1H), 4.0–4.0 (m, 1H), 3.1–3.2 (m, 2H), 3.0–3.0 (m, 1H), 2.8–2.9 (m, 2H), 2.63 (br dd, 1H, *J =* 4.9, 15.9 Hz), 1.4–1.6 (m, 9H), 0.8–0.9 (m, 18H); ^13^C NMR (DMSO-*d_6_*, 101 MHz) *δ* 173.6, 172.1, 172.0, 171.9, 171.1, 170.8, 169.6, 136.1, 133.5, 129.3, 126.9, 123.4, 120.9, 118.4, 118.1, 116.8, 111.3, 109.8, 55.8, 52.4, 52.3, 51.1, 50.4, 49.5, 41.8, 40.4, 36.4, 25.9, 25.8, 24.6, 24.2, 24.1, 23.1, 22.6, 22.4, 22.3, 22.1, 20.6. (+)-HRESIMS *m/z* 768.4768 [M + H]^+^ (calcd for C_39_H_62_N_9_O_7_, 768.4767).

Desotamide A0 (**19**): cyclo(Asn-Ile-d-Leu-Leu-Trp-d-Arg). ^1^H NMR (400 MHz, DMSO-*d6*) *δ* ppm 0.79–0.93 (m, 18 H) 1.32–1.68 (m, 13 H) 2.65 (qd, J = 15.64, 6.02 Hz, 4 H) 2.96 (br dd, J = 14.56, 10.29 Hz, 1 H) 3.18 (br dd, J = 14.62, 4.08 Hz, 1 H) 4.02 (ddd, J = 10.63, 6.81, 4.14 Hz, 1 H) 4.11–4.19 (m, 2 H) 4.27–4.35 (m, 1 H) 4.44 (q, J = 7.11 Hz, 1 H) 4.54–4.60 (m, 1 H) 6.96–7.01 (m, 1 H) 7.01–7.04 (m, 1 H) 7.04–7.09 (m, 1 H) 7.13 (d, J = 1.88 Hz, 1 H) 7.33 (d, J = 8.03 Hz, 1 H) 7.44 (br d, J = 8.16 Hz, 1 H) 7.51 (br d, J = 5.40 Hz, 1 H) 7.53 (br d, J = 5.14 Hz, 1 H) 7.59 (br s, 1 H) 7.61 (br s, 1 H) 7.65 (br s, 2 H) 8.21–8.28 (m, 1 H) 8.41 (br d, J = 5.27 Hz, 1 H) 8.70 (br d, J = 7.03 Hz, 1 H) 10.84 (d, J = 1.25 Hz, 1 H);^13^C NMR (DMSO-*d_6_*, 101 MHz) *δ* 173.0, 171.9, 171.8, 171.6, 171.0, 170.8, 170.6, 136.1, 127.0, 123.6, 120.9, 118.4, 118.1, 111.3, 110.0, 55.3, 52.2, 51.9, 51.8, 50.5, 49.4, 41.9, 40.4, 38.2, 37.1, 27.2, 26.5, 25.9, 25.8, 24.5, 24.2, 24.1, 23.2, 23.2, 22.6, 22.5, 22.4, 22.1, 20.6. (+)-HRESIMS *m/z* 777.4407 [M + H]^+^ (calcd for C_39_H_57_N_10_O_7_, 777.4406). 

### 3.3. Antibacterial Activities

The MIC values of compounds **1** and **7**–**19** were determined against all the pathogens tested by sequential two-fold serial dilutions technique using Mueller-Hinton broth in 96-well plate according to the previously reported method [17], which is based on the method by the Clinical and Laboratory Standards Institute (CLSI) [18]. Ampicillin, vancomycin and polymyxin were used as control. The maximum test concentrations for all the compounds were 128 μg/mL.

## 4. Conclusions

In summary, we have synthesized 10 analogues of desotamide A (**1**), natural products wollamide B and its two analogues, and evaluated their antibacterial activities against a panel of Gram-positive and -negative bacteria pathogens. This study revealed that residues at position II and VI greatly impacted antibacterial activity. Among the 10 synthesized desotamide analogues, two [desotamide A4 (**13**) and desotamide A6 (**15**)] showed a 2–4-fold increase of antibacterial activities with MIC values of 8–32 μg/mL compared to the original cyclopeptide desotamide A (**1**). Significantly, compounds **13** and **15** were found to exhibit activities against several clinical isolates of MRSA, which is one of the most prevalent causes of nosocomial infections and presents a significant challenge for treatment with current clinic antibiotics. The enhanced antibacterial activity, a broad antibacterial spectrum of compounds **13** and **15** highlighted their potential as new antibiotic leads for further development.

## Abbreviation:

MRSE: methicillin-resistant *Staphylococcus epidermidis*; MRSA: methicillin-resistant *Staphylococcus aureus*; VRE: vancomycin-resistant *Enterococcus*; SAR: structure-activity relationship; 2-CTC: 2-chlorotrityl chloride; DMF: *N*,*N*-dimethylformamide; DIEA: *N*,*N*-diisopropylethylamine; TFA: trifluoroacetic acid; DCM: dichloromethane; HBTU: 2-(1H-Benzotriazole-1-yl)-1,1,3,3-tetramethyluronium hexafluorophosphate; MIC: minimum inhibitory concentration; CLSI: Clinical and Laboratory Standards Institute; RP-HPLC: reversed-phase high performance liquid chromatogram.

## Figures and Tables

**Scheme 1:**
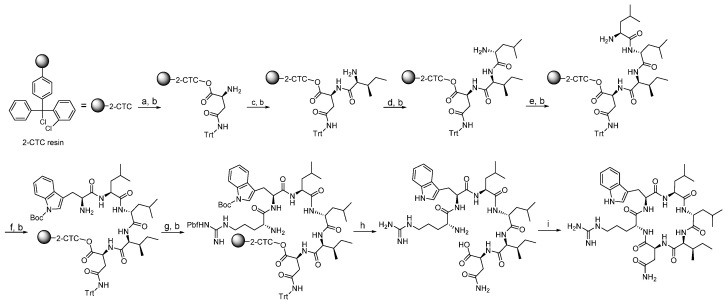
Synthesis of desotamide A1 on 2-chlorotrityl chloride (2-CTC) resin. Reagents and conditions: (**a**) Fmoc-l-Asn(trt)-OH, HBTU, DIEA, DMF, 1.5 h; (**b**) 20% piperidine in DMF, 0.25 h; (**c**) Fmoc-l-*allo*-Ile-OH, HBTU, DIEA, DMF, 0.5 h; (**d**) Fmoc-d-Leu-OH, HBTU, DIEA, DMF, 0.5 h; (**e**) Fmoc-l-Leu-OH, HBTU, DIEA, DMF, 0.5 h; (**f**) Fmoc-l-Trp(Boc)-OH, HBTU, DIEA, DMF, 0.5 h; (**g**) Fmoc-d-Arg(Pbf)-OH, HBTU, DIEA, DMF, 0.5 h; (**h**) TFA/thioanisole/phenol/dithioglycol/H_2_O 33/2/2/1/2, 2 h; (**i**) HBTU, DIEA, pH 8.0–9.0.

**Table 1:** Antibacterial activities (MIC, μg/mL) cyclic hexapeptides **1** and **7–19**.

	Compounds No.																
Pathogens	1	10	11	12	13	14	15	16	17	18	19	7	8	9	Amp ^a^	Van ^b^	Pol ^c^
*Staphylococcus simulans* LJH13	16	>128	>128	>128	8	64	8	>128	32	>128	32	16	8	16	0.25	0.25	-
*Bacillus subtilis* BS01	32	>128	>128	>128	8	64	8	>128	32	>128	32	32	8	16	<0.0625	0.125	-
*Micrococcus luteus* ML01	32	64	>128	>128	8	64	8	>128	16	>128	32	16	8	16	0.25	0.25	-
*Enterococcus gallinarum* LJH11	64	>128	>128	>128	64	128	128	>128	>128	>128	>128	>128	64	128	2	8	-
*Staphylococcus haemolyticus* LJH9	128	>128	>128	>128	32	64	32	>128	64	>128	64	64	32	32	16	2	-
*Enterococcus faecalis* ATCC 29212	64	>128	>128	>128	32	64	32	>128	128	>128	128	128	32	64	4	4	-
*Staphylococcus aureus* ATCC 29213	32	>128	>128	>128	16	64	16	>128	32	>128	64	32	16	32	1	0.5	-
methicillin-resistant *Staphylococcus aureus* (MRSA) shhsE1	64	>128	>128	>128	16	64	8	>128	64	>128	64	32	16	32	64	0.5	-
MRSA 16339	16	>128	>128	>128	16	64	16	>128	64	>128	128	64	16	32	64	0.25	-
MRSA 745524	8	>128	>128	>128	16	64	8	>128	32	>128	64	32	16	32	16	0.5	-
MRSA 16162	32	>128	>128	>128	16	64	16	>128	64	>128	64	64	16	32	32	0.5	-
*E. coli* ATCC 13124	>128	>128	>128	>128	64	>12	128	>128	>128	>128	>128	128	128	128	-	-	16
*Klebsiella pneumoniae* 15580	>128	>128	>128	>128	>128	>128	>128	>128	>128	>128	>128	>128	>128	>128	-	-	2

“-”: not tested; a: ampicillin; b: vancomycin; c: polymyxin.

## Data Availability

The authors declare that all relevant data supporting the findings of this study are available within the article and its Supplementary Materials file, or from the corresponding authors upon request.

## Supplementary Materials

The following are available online at https://www.mdpi.com/article/10.3390/md19060303/s1. Figures S1–S39: The HRESIMS, 1 D NMR spectra for compounds **7–19**.
