# Supplementary material for: Chemical Synthesis and Structure-Activity Relationship Study Yield Desotamide a Analogues with Improved Antibacterial Activity
Source: Mar Drugs. 2021 May 24;19(6):303. doi: 10.3390/md19060303 (PMC8225045; doi:10.3390/md19060303)

marine drugs

MDPI

*Communication*

# Chemical Synthesis and Structure-Activity Relationship Study Yield Desotamide A Analogues with Improved Antibacterial Activity

**Run xu [1,3,‡], Yongxiang Song [1,‡], Jun Li [1], Jianhua Ju [1,2], and Qinglian Li [1,*]**

[1] CAS Key Laboratory of Tropical Marine Bio-resources and Ecology, Guangdong Key Laboratory of Marine Materia Medica, RNAM Center for Marine Microbiology, South China Sea Institute of Oceanology, Chinese Academy of Sciences, 164 West Xingang Rd., Guangzhou 510301, China; runxu126@126.com (R.X.); songx@scsio.ac.cn (Y.S.); lijun@scsio.ac.cn (J.L.); jju@scsio.ac.cn (J.J.)
[2] College of Oceanology, University of Chinese Academy of Sciences, 19 Yuquan Road, Beijing 100049, China
[3] School of Pharmacy, Zunyi Medical University, 201 Dalian Road, Zunyi 563000, China
* Correspondence: liql@scsio.ac.cn; Tel./Fax: +86-20-3406-6449
‡ These authors contributed equally to this work.

| Table of contents | Page |
|---|---|
| **Figures S1-S3**. HRESIMS, and 1D NMR spectra in DMSO-$d_6$ of **7** | S2-S4 |
| **Figures S4-S6**. HRESIMS, and 1D NMR spectra in DMSO-$d_6$ of **8** | S5-S7 |
| **Figures S7-S9**. HRESIMS, and 1D NMR spectra in DMSO-$d_6$ of **9** | S8-S10 |
| **Figures S10-S12**. HRESIMS, and 1D NMR spectra in DMSO-$d_6$ of **10** | S11-S13 |
| **Figures S13-S15**. HRESIMS, and 1D NMR spectra in DMSO-$d_6$ of **11** | S14-S16 |
| **Figures S16-S18**. HRESIMS, and 1D NMR spectra in DMSO-$d_6$ of **12** | S17-S19 |
| **Figures S19-S21**. HRESIMS, and 1D NMR spectra in DMSO-$d_6$ of **13** | S20-S22 |
| **Figures S22-S24**. HRESIMS, and 1D NMR spectra in DMSO-$d_6$ of **14** | S23-S25 |
| **Figures S25-S27**. HRESIMS, and 1D NMR spectra in DMSO-$d_6$ of **15** | S26-S28 |
| **Figures S28-S30**. HRESIMS, and 1D NMR spectra in DMSO-$d_6$ of **16** | S29-S31 |
| **Figures S31-S33**. HRESIMS, and 1D NMR spectra in DMSO-$d_6$ of **17** | S32-S34 |
| **Figures S34-S36**. HRESIMS, and 1D NMR spectra in DMSO-$d_6$ of **18** | S35-S37 |
| **Figures S7-S39**. HRESIMS, and 1D NMR spectra in DMSO-$d_6$ of **19** | S38-S40 |

Figures S1. The HRESIMS spectrum of **7**

## Mass Spectrum SmartFormula Report

**Analysis Info**

| | | | |
|---|---|---|---|
| Analysis Name | D:\Data\MS\data\202102\liqinglian_B_pos_46_01_10003.d | Acquisition Date | 2/8/2021 3:17:21 PM |
| Method | LC_Direct Infusion_pos_100-1000mz.m | Operator | SCSIO |
| Sample Name | liqinglian_B_pos | Instrument | maXis 255552.00029 |
| Comment | | | |

**Acquisition Parameter**

| | | | | | |
|---|---|---|---|---|---|
| Source Type | ESI | Ion Polarity | Positive | Set Nebulizer | 0.4 Bar |
| Focus | Active | Set Capillary | 4500 V | Set Dry Heater | 180 °C |
| Scan Begin | 100 m/z | Set End Plate Offset | -500 V | Set Dry Gas | 4.0 l/min |
| Scan End | 2000 m/z | Set Charging Voltage | 0 V | Set Divert Valve | Waste |
| | | Set Corona | 0 nA | Set APCI Heater | 0 °C |

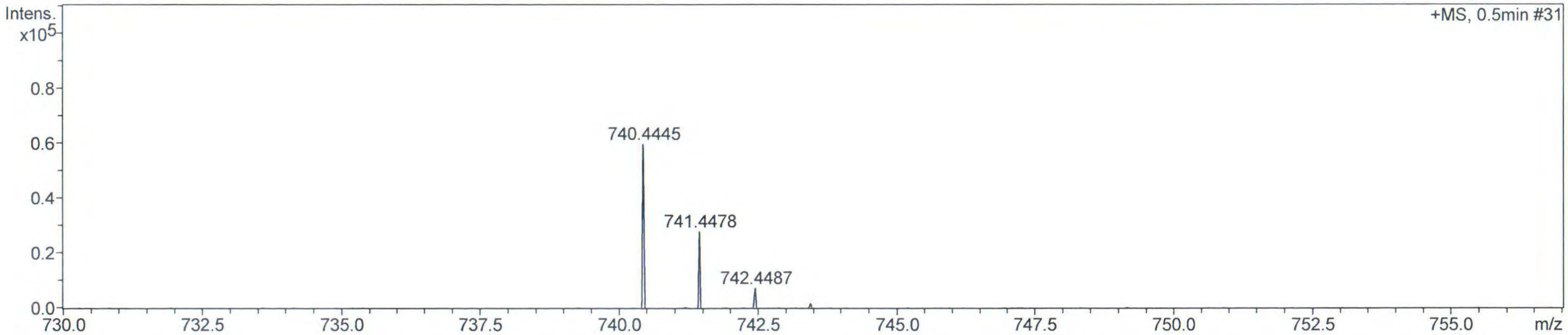


| Meas. m/z | # | Ion Formula | Score | m/z | err [ppm] | err [mDa] | mSigma | rdb | e⁻ Conf | N-Rule |
|---|---|---|---|---|---|---|---|---|---|---|
| 740.4445 | 1 | C37H58N9O7 | 100.00 | 740.4454 | 1.2 | 0.9 | 15.8 | 13.5 | even | ok |

liqinglian_B_pos_46_01_10003.d

Bruker Compass DataAnalysis 4.1 printed: 2/8/2021 3:21:17 PM by: SCSIO Page 1 of 1

Figures S2. The 1H NMR spectrum of **7** in DMSO-$d_6$

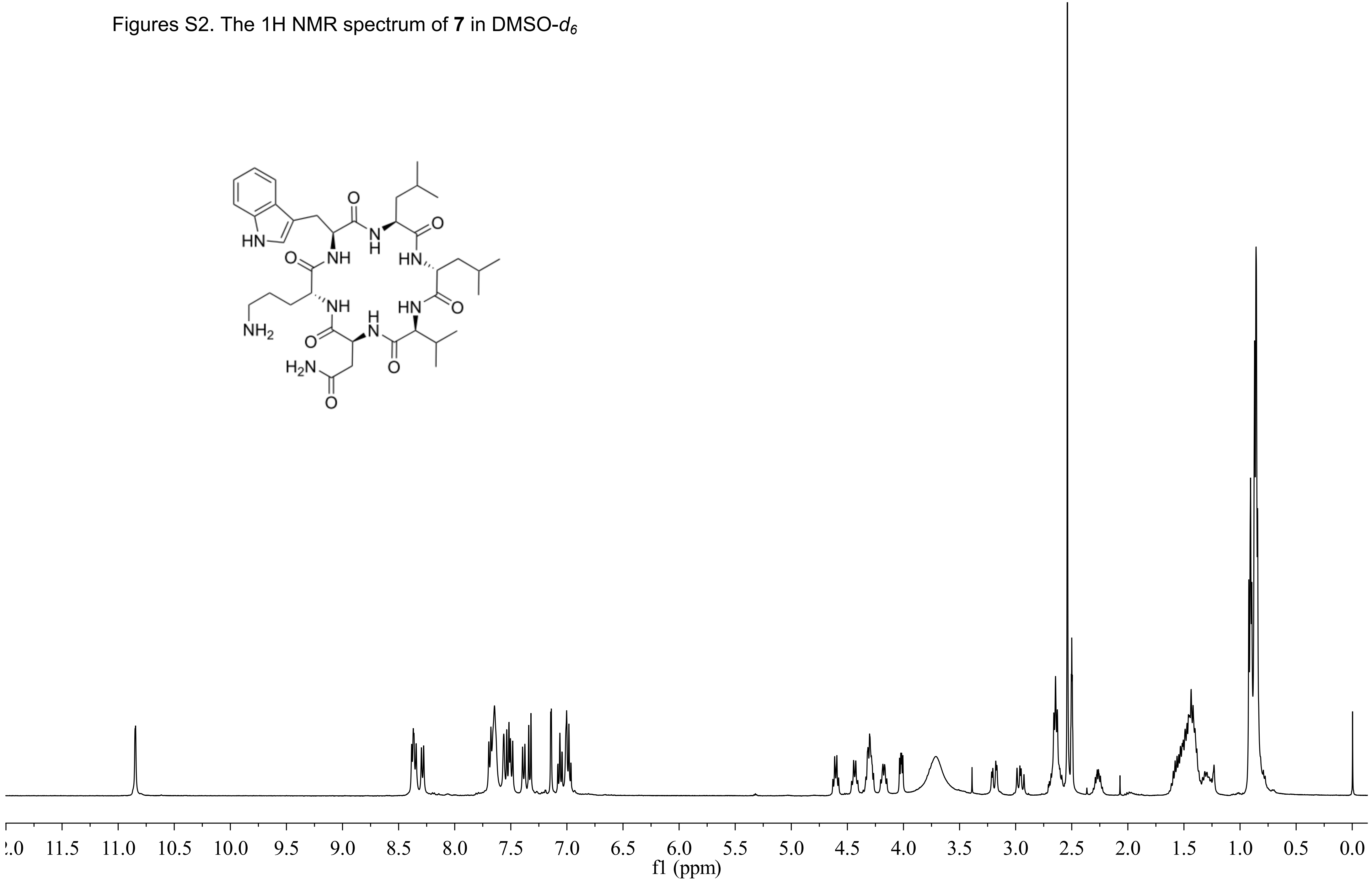

Figures S3. The $^{13}C$ NMR spectrum of **7** in DMSO-$d_6$

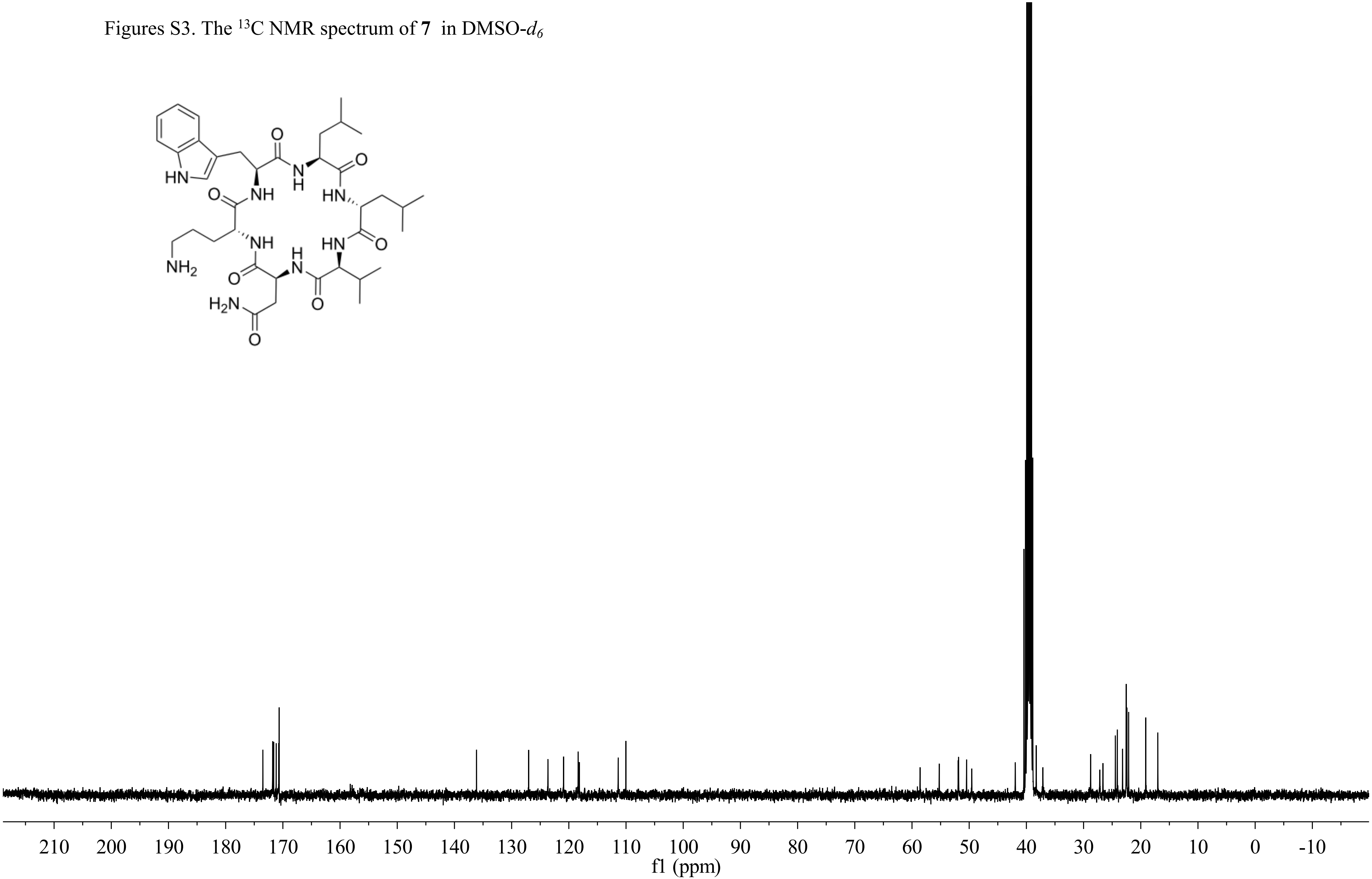

Figures S4. The HRESIMS spectrum of **8**

# Mass Spectrum SmartFormula Report

**Analysis Info**

| | | | |
|---|---|---|---|
| Analysis Name | D:\Data\MS\data\202102\liqinglian_B1_pos_47_01_10004.d | Acquisition Date | 2/8/2021 3:20:48 PM |
| Method | LC_Direct Infusion_pos_100-1000mz.m | Operator | SCSIO |
| Sample Name | liqinglian_B1_pos | Instrument | maXis 255552.00029 |
| Comment | | | |

**Acquisition Parameter**

| | | | | | |
|---|---|---|---|---|---|
| Source Type | ESI | Ion Polarity | Positive | Set Nebulizer | 0.4 Bar |
| Focus | Active | Set Capillary | 4500 V | Set Dry Heater | 180 °C |
| Scan Begin | 100 m/z | Set End Plate Offset | -500 V | Set Dry Gas | 4.0 l/min |
| Scan End | 2000 m/z | Set Charging Voltage | 0 V | Set Divert Valve | Waste |
| | | Set Corona | 0 nA | Set APCI Heater | 0 °C |

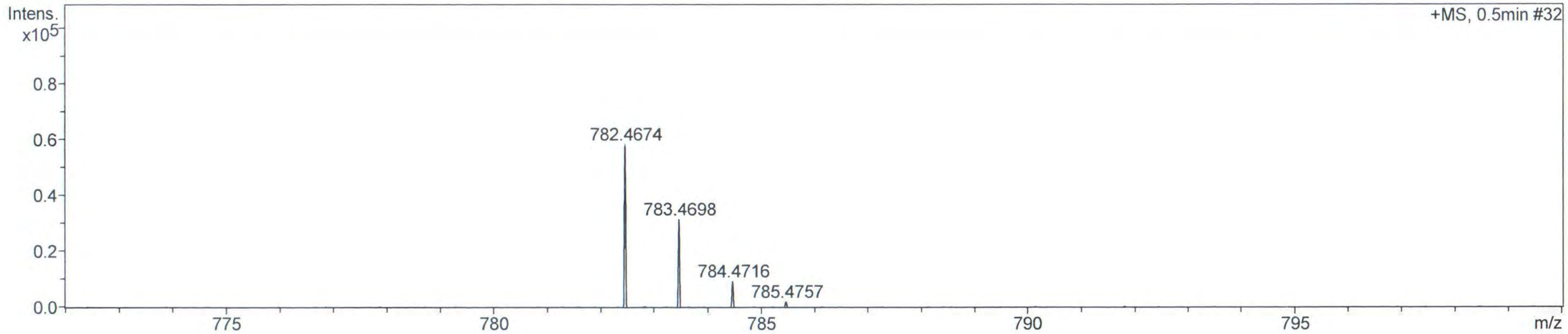


| Meas. m/z | # | Ion Formula | Score | m/z | err [ppm] | err [mDa] | mSigma | rdb | e¯ Conf | N-Rule |
|---|---|---|---|---|---|---|---|---|---|---|
| 782.4674 | 1 | C38H60N11O7 | 100.00 | 782.4672 | -0.3 | -0.2 | 45.4 | 14.5 | even | ok |

liqinglian_B1_pos_47_01_10004.d
Bruker Compass DataAnalysis 4.1 printed: 2/8/2021 3:25:35 PM by: SCSIO Page 1 of 1

Figures S5. The $^{1}$H NMR spectrum of **8** in DMSO-$d_6$

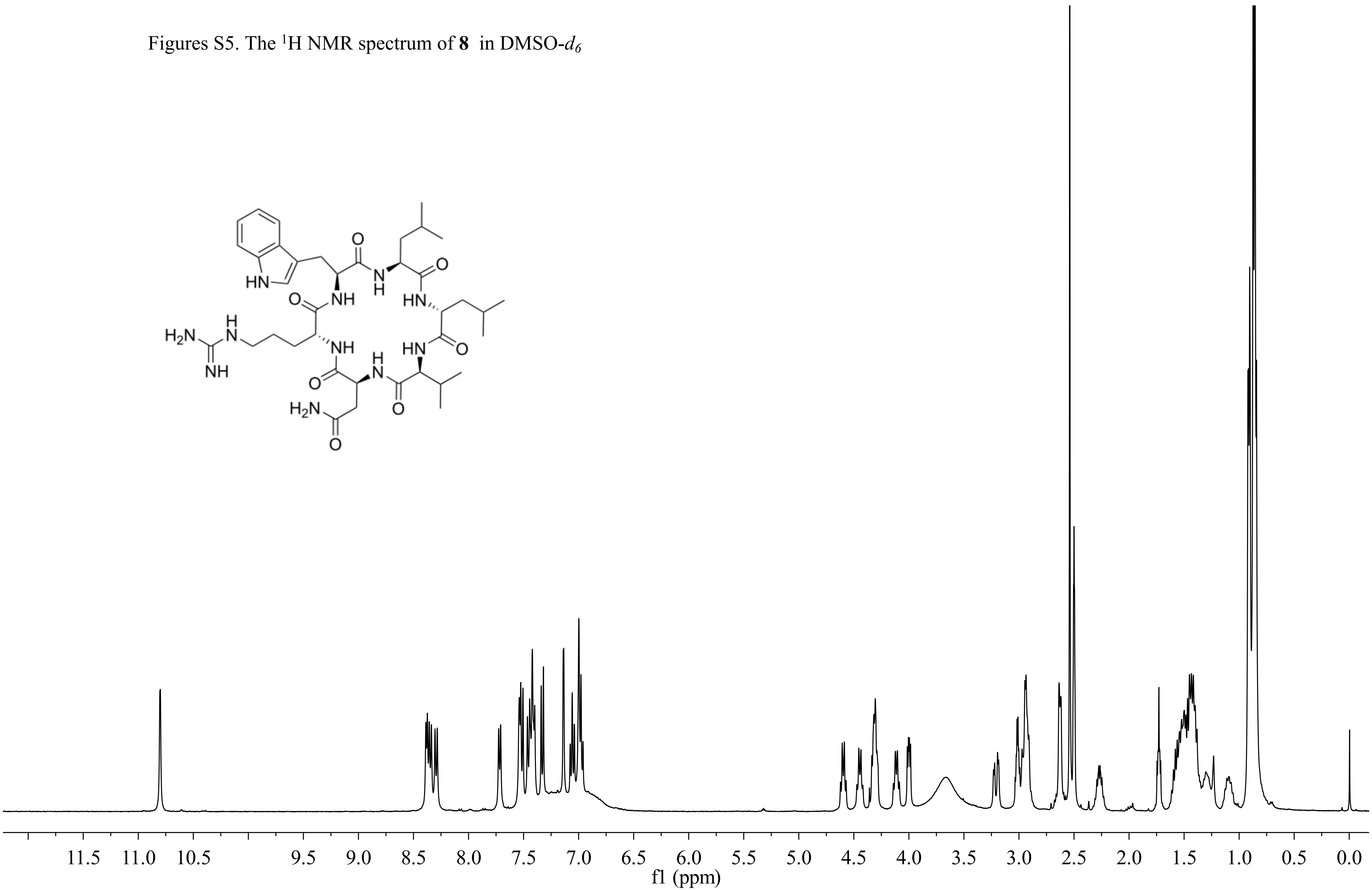

Figures S6. The $^{13}$C NMR spectrum of **8** in DMSO-$d_6$

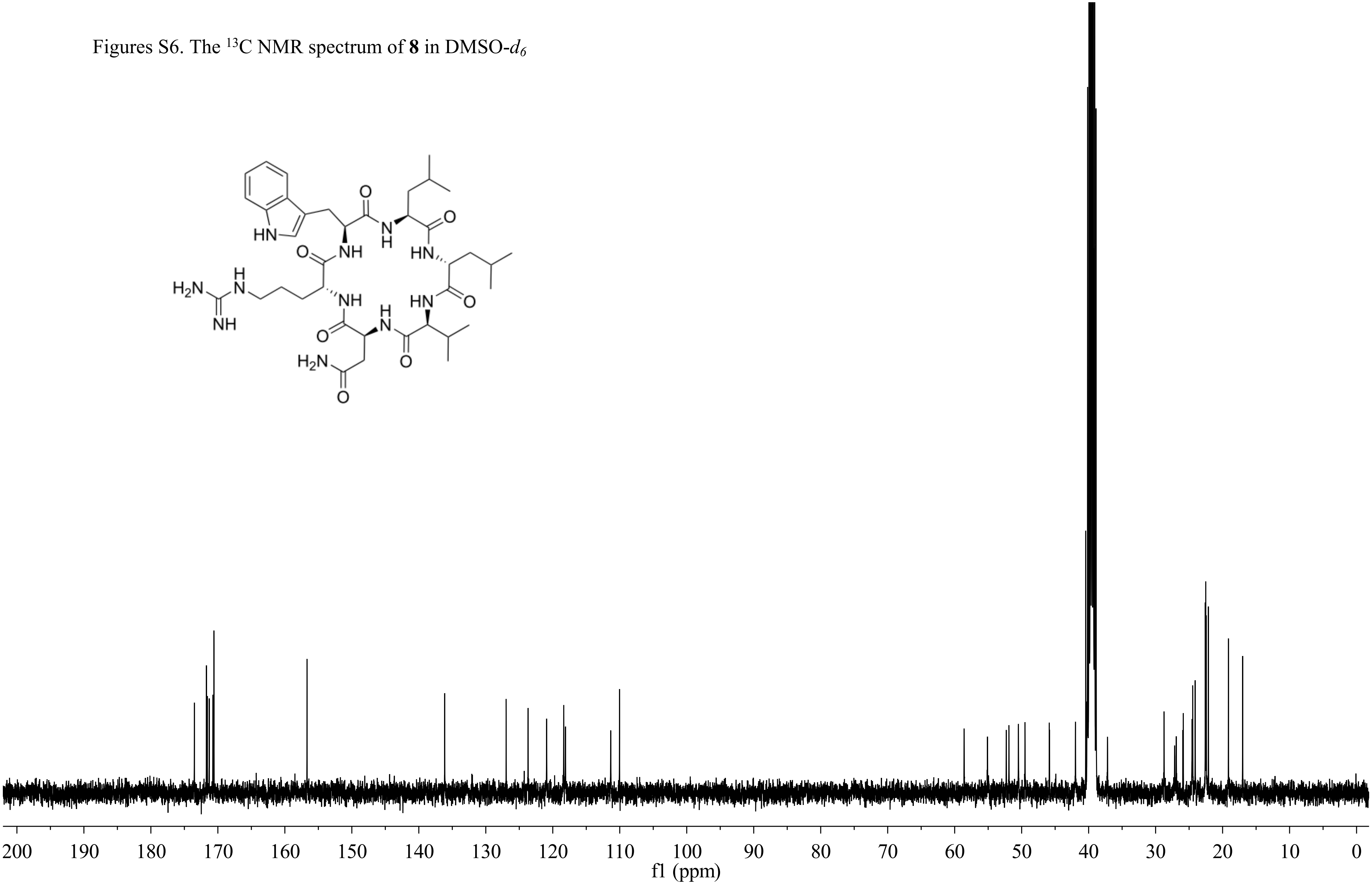

Figures S7. The HRESIMS spectrum of **9**

# Mass Spectrum SmartFormula Report

**Analysis Info**

| | | | | |
|---|---|---|---|---|
| Analysis Name | D:\Data\MS\data\202102\liqinglian_B2_pos_48_01_10005.d | Acquisition Date | 2/8/2021 3:24:16 PM | |
| Method | LC_Direct Infusion_pos_100-1000mz.m | Operator | SCSIO | |
| Sample Name | liqinglian_B2_pos | Instrument | maXis | 255552.00029 |
| Comment | | | | |

**Acquisition Parameter**

| | | | | | |
|---|---|---|---|---|---|
| Source Type | ESI | Ion Polarity | Positive | Set Nebulizer | 0.4 Bar |
| Focus | Active | Set Capillary | 4500 V | Set Dry Heater | 180 °C |
| Scan Begin | 100 m/z | Set End Plate Offset | -500 V | Set Dry Gas | 4.0 l/min |
| Scan End | 2000 m/z | Set Charging Voltage | 0 V | Set Divert Valve | Waste |
| | | Set Corona | 0 nA | Set APCI Heater | 0 °C |

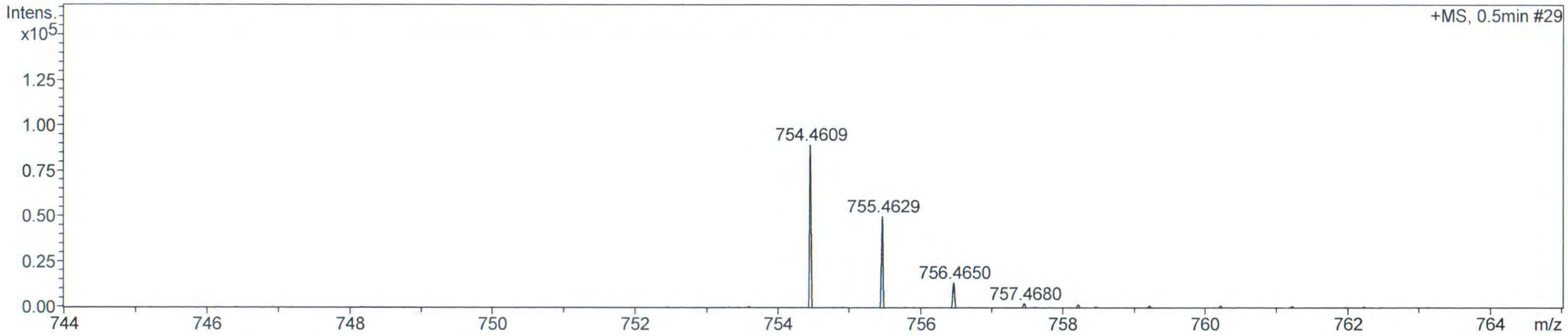


| Meas. m/z | # | Ion Formula | Score | m/z | err [ppm] | err [mDa] | mSigma | rdb | e⁻ Conf | N-Rule |
|---|---|---|---|---|---|---|---|---|---|---|
| 754.4609 | 1 | C38H60N9O7 | 100.00 | 754.4610 | -0.1 | -0.1 | 57.0 | 13.5 | even | ok |

liqinglian_B2_pos_48_01_10005.d
Bruker Compass DataAnalysis 4.1 printed: 2/8/2021 3:28:45 PM by: SCSIO Page 1 of 1

Figures S8. The $^{1}$H NMR spectrum of **9** in DMSO-$d_6$

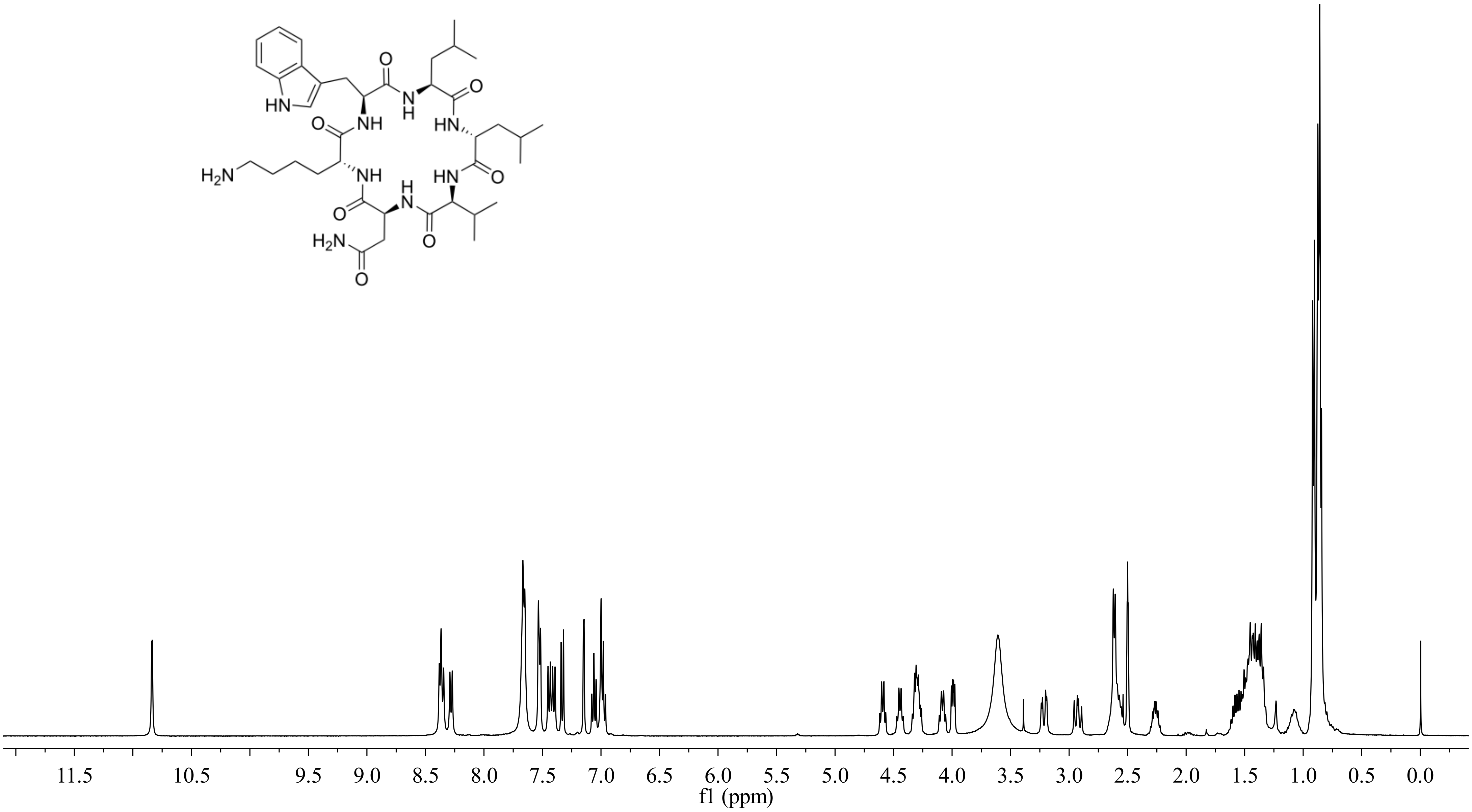

Figures S9. The $^{13}C$ NMR spectrum of **9** in DMSO-$d_6$

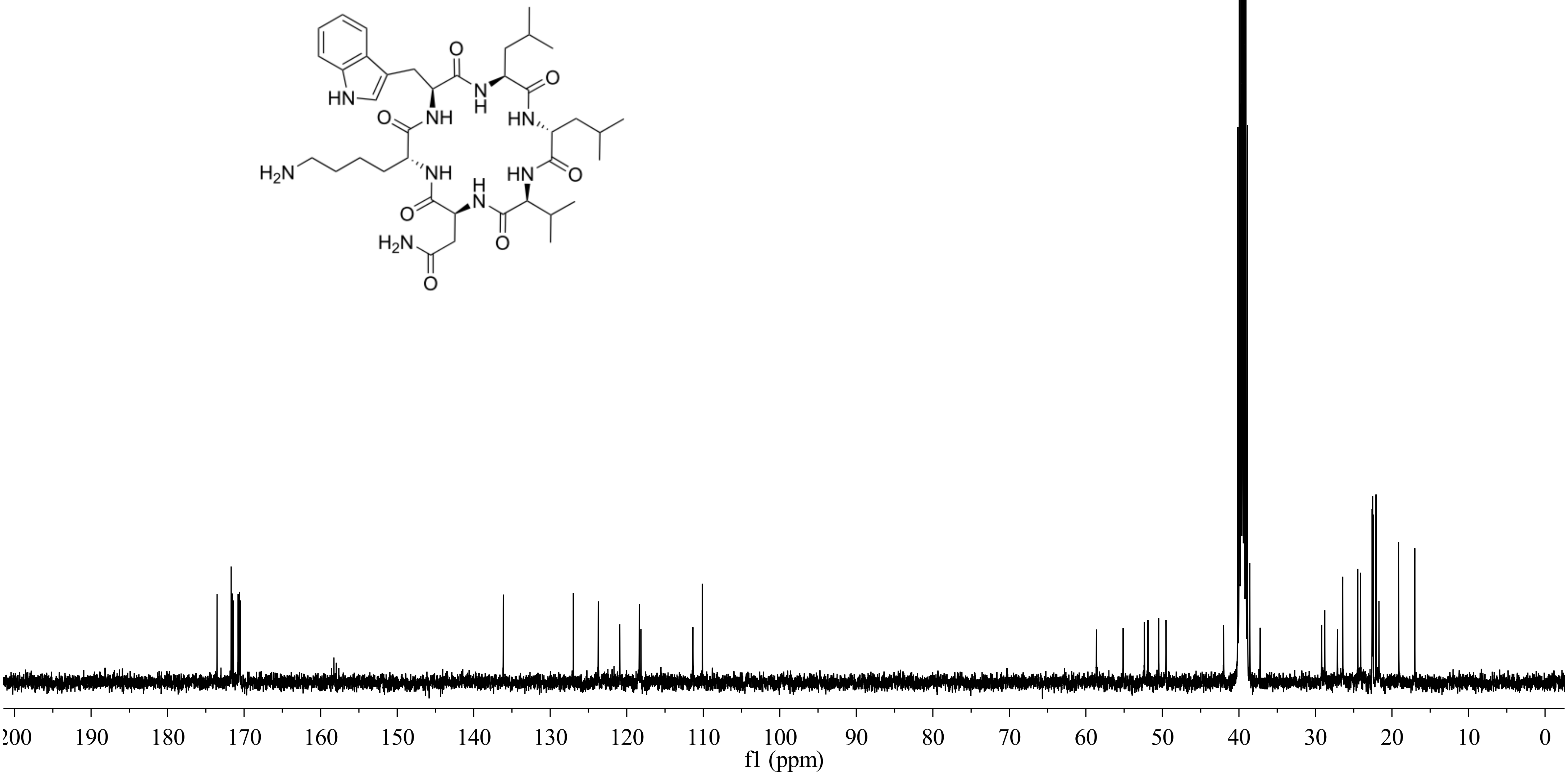

Figures S10. The HRESIMS spectrum of **10**

## Mass Spectrum SmartFormula Report

**Analysis Info**

| | | | |
|---|---|---|---|
| Analysis Name | D:\Data\MS\data\202102\liqinglian_A1_pos_52_01_9993.d | Acquisition Date | 2/8/2021 2:42:35 PM |
| Method | LC_Direct Infusion_pos_100-1000mz.m | Operator | SCSIO |
| Sample Name | liqinglian_A1_pos | Instrument | maXis 255552.00029 |
| Comment | | | |

**Acquisition Parameter**

| | | | | | |
|---|---|---|---|---|---|
| Source Type | ESI | Ion Polarity | Positive | Set Nebulizer | 0.4 Bar |
| Focus | Active | Set Capillary | 4500 V | Set Dry Heater | 180 °C |
| Scan Begin | 100 m/z | Set End Plate Offset | -500 V | Set Dry Gas | 4.0 l/min |
| Scan End | 2000 m/z | Set Charging Voltage | 0 V | Set Divert Valve | Waste |
| | | Set Corona | 0 nA | Set APCI Heater | 0 °C |

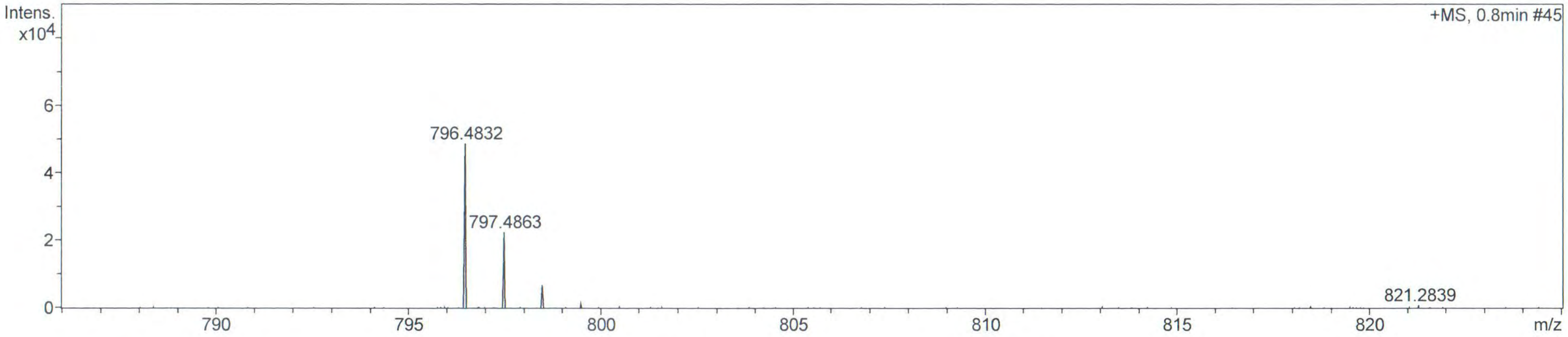


| Meas. m/z | # | Ion Formula | Score | m/z | err [ppm] | err [mDa] | mSigma | rdb | e⁻ Conf | N-Rule |
|---|---|---|---|---|---|---|---|---|---|---|
| 796.4832 | 1 | C39H62N11O7 | 100.00 | 796.4828 | 0.4 | 0.3 | 11.4 | 14.5 | even | ok |

liqinglian_A1_pos_52_01_9993.d
Bruker Compass DataAnalysis 4.1 printed: 2/8/2021 2:47:21 PM by: SCSIO Page 1 of 1

Figures S11. The 1H NMR spectrum of 10 in DMSO-$d_6$

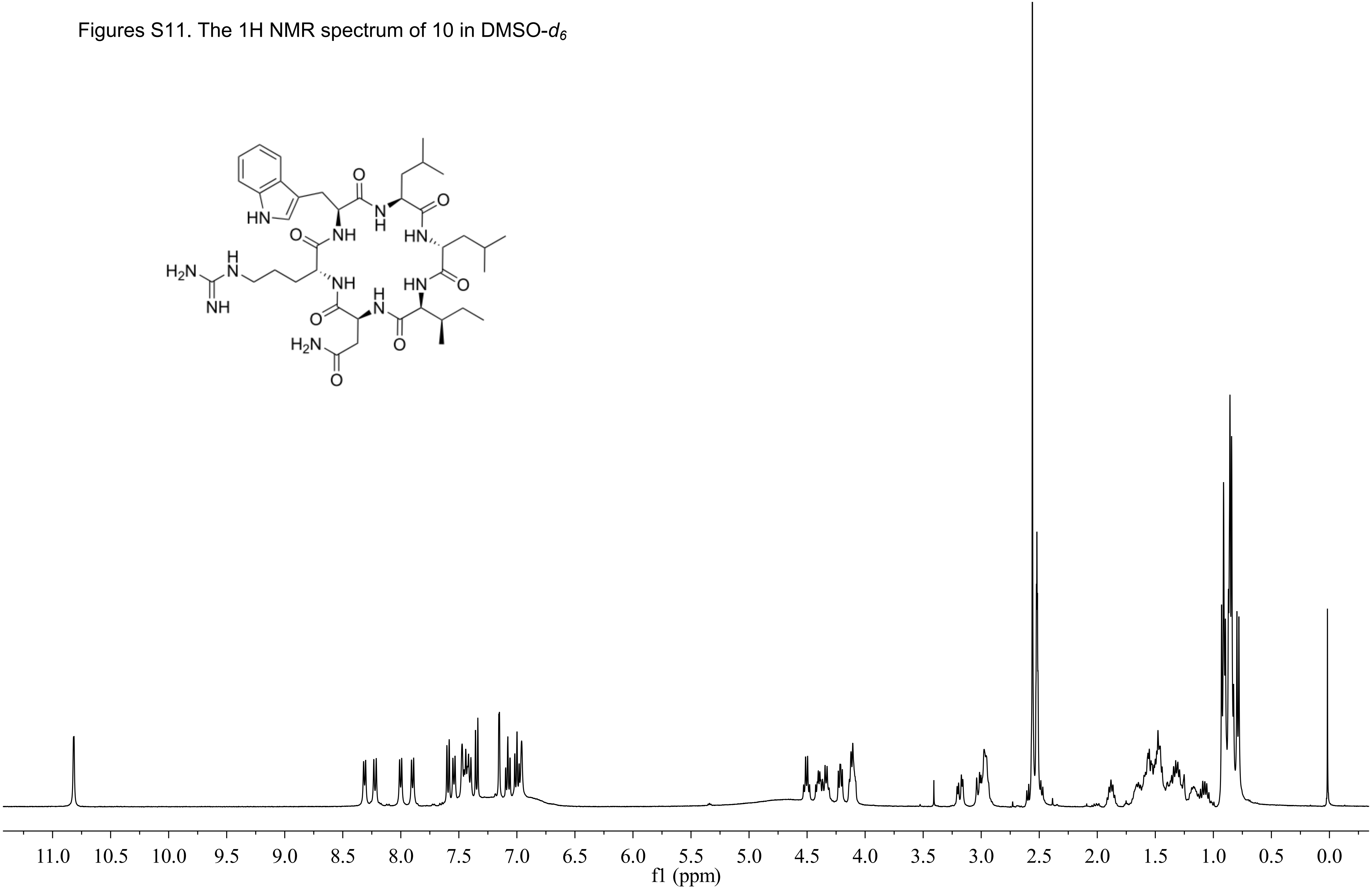

Figures S12. The 13C NMR spectrum of 10 in DMSO-$d_6$

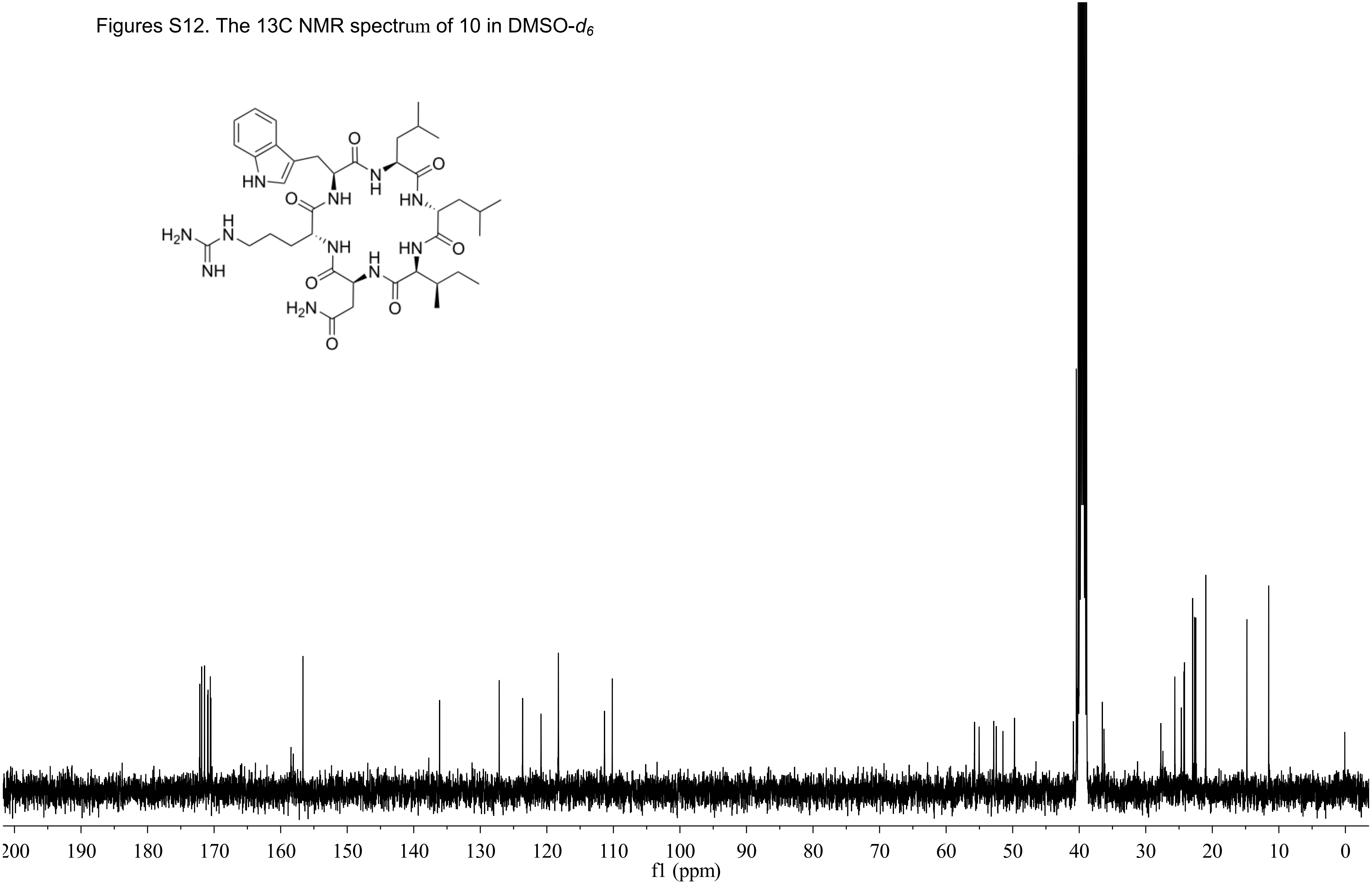

Figures S13. The HRESIMS spectrum of **11**

# Mass Spectrum SmartFormula Report

**Analysis Info**

| | | | |
|---|---|---|---|
| Analysis Name | D:\Data\MS\data\202102\liqinglian_A2_pos_53_01_9994.d | Acquisition Date | 2/8/2021 2:46:04 PM |
| Method | LC_Direct Infusion_pos_100-1000mz.m | Operator | SCSIO |
| Sample Name | liqinglian_A2_pos | Instrument | maXis 255552.00029 |
| Comment | | | |

**Acquisition Parameter**

| | | | | | |
|---|---|---|---|---|---|
| Source Type | ESI | Ion Polarity | Positive | Set Nebulizer | 0.4 Bar |
| Focus | Active | Set Capillary | 4500 V | Set Dry Heater | 180 °C |
| Scan Begin | 100 m/z | Set End Plate Offset | -500 V | Set Dry Gas | 4.0 l/min |
| Scan End | 2000 m/z | Set Charging Voltage | 0 V | Set Divert Valve | Waste |
| | | Set Corona | 0 nA | Set APCI Heater | 0 °C |

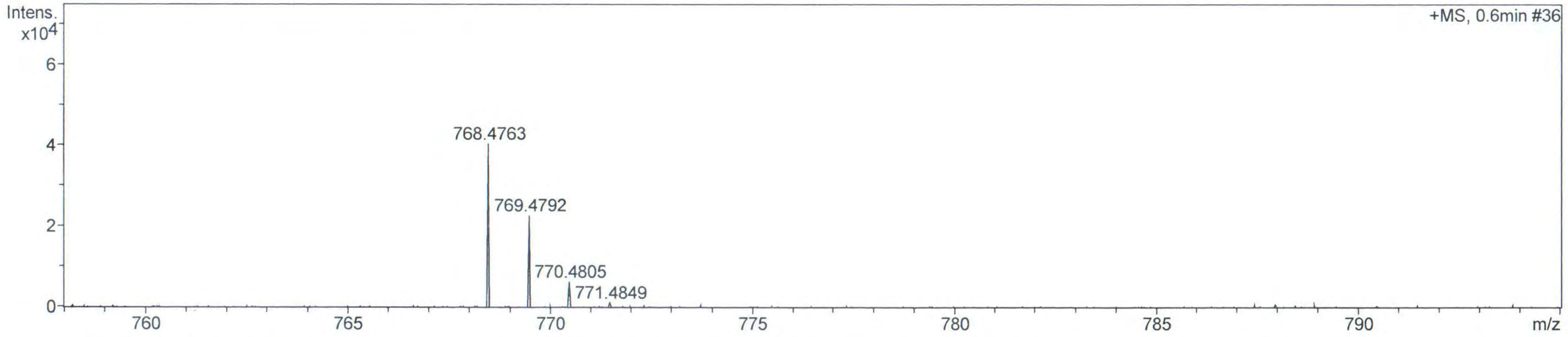


| Meas. m/z | # | Ion Formula | Score | m/z | err [ppm] | err [mDa] | mSigma | rdb | e⁻ Conf | N-Rule |
|---|---|---|---|---|---|---|---|---|---|---|
| 768.4763 | 1 | C39H62N9O7 | 100.00 | 768.4767 | 0.5 | 0.4 | 50.8 | 13.5 | even | ok |

liqinglian_A2_pos_53_01_9994.d

Bruker Compass DataAnalysis 4.1 printed: 2/8/2021 2:50:57 PM by: SCSIO Page 1 of 1

Figures S14. The $^{1}$H NMR spectrum of **11** in DMSO-$d_6$

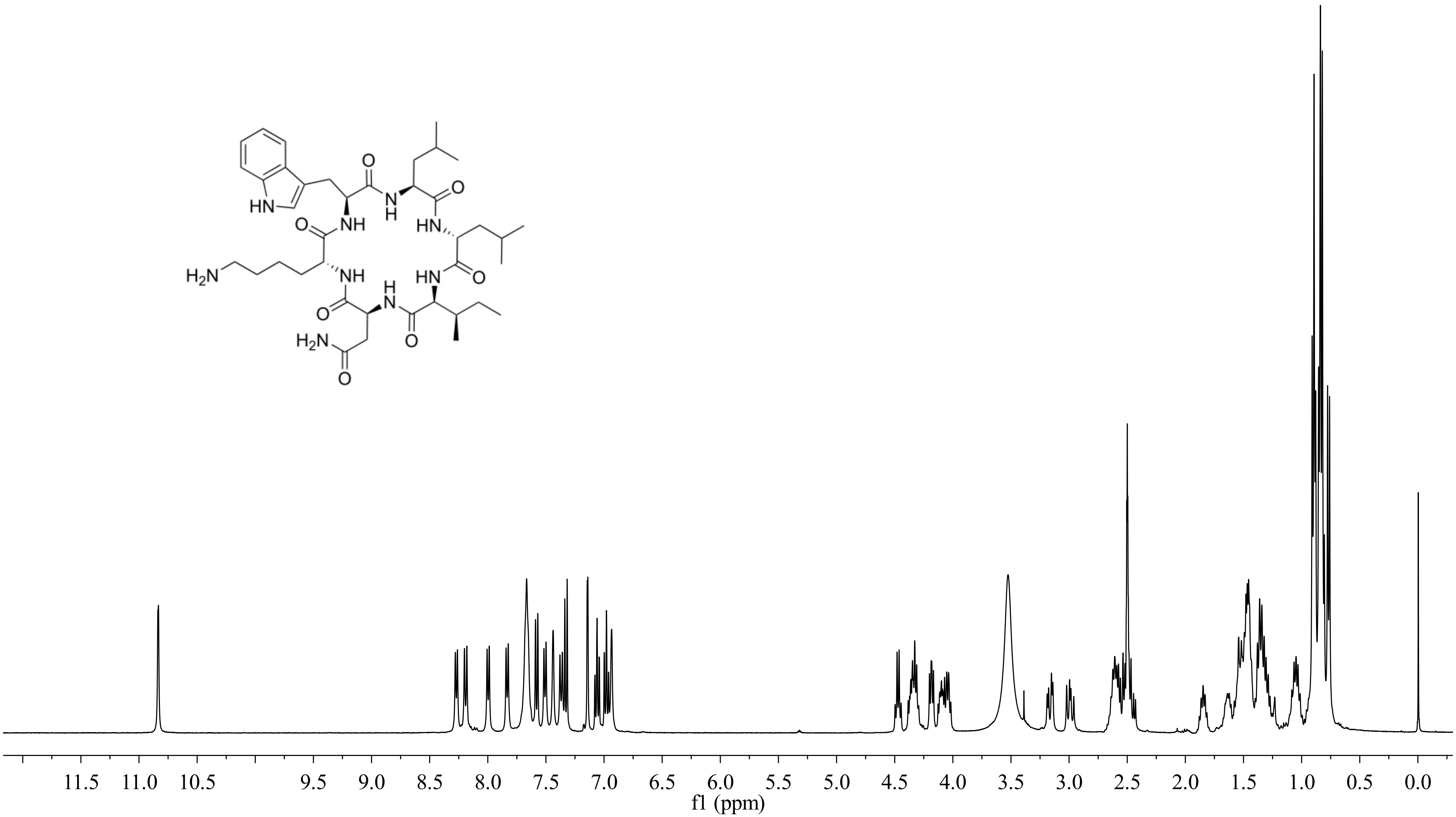

Figures S15. The 13C NMR spectrum of 11 in DMSO-$d_6$

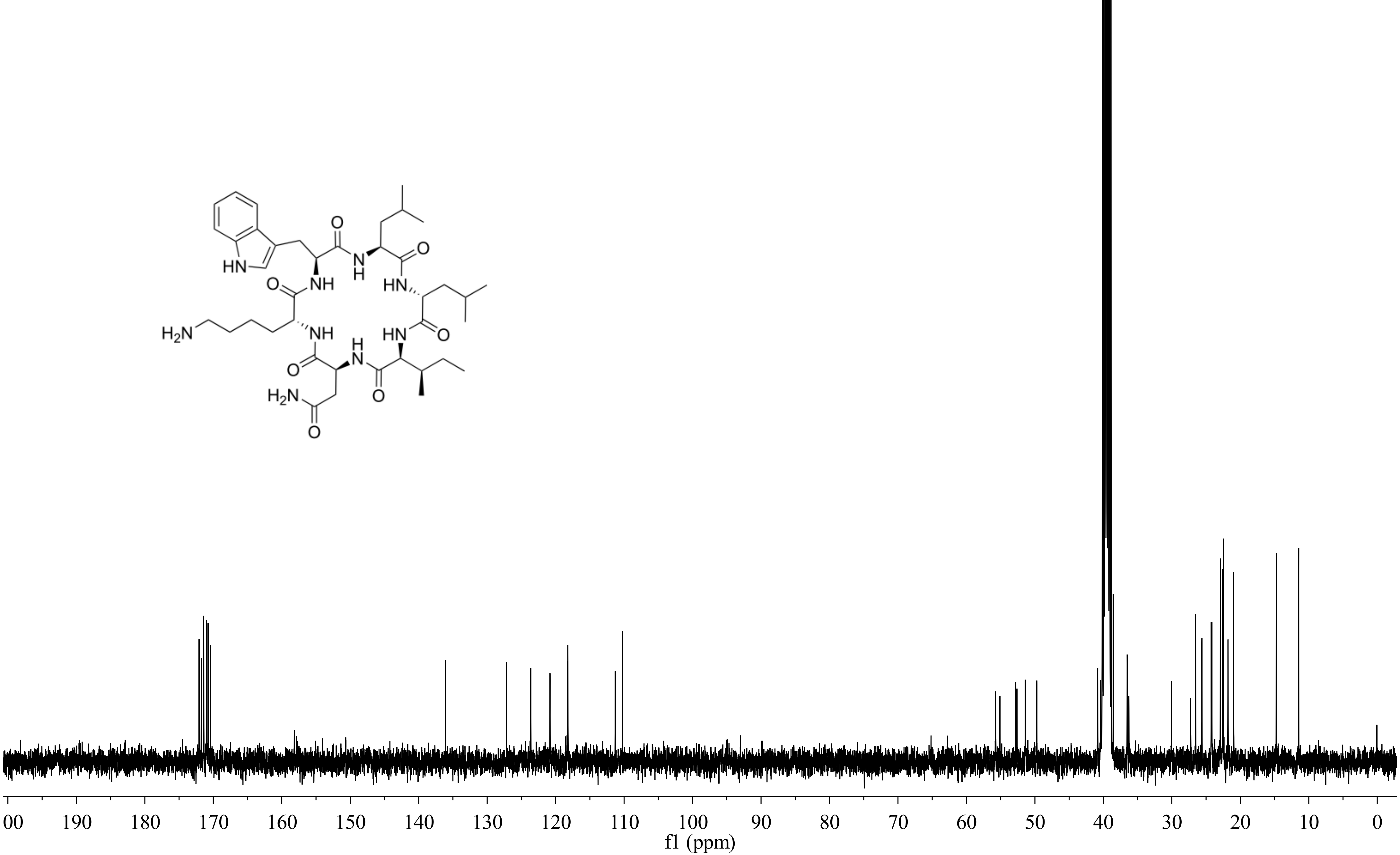

Figures S16. The HRESIMS spectrum of **12**

## Mass Spectrum SmartFormula Report

**Analysis Info**

| | | | |
|---|---|---|---|
| Analysis Name | D:\Data\MS\data\202102\liqinglian_A3_pos_54_01_9995.d | Acquisition Date | 2/8/2021 2:49:33 PM |
| Method | LC_Direct Infusion_pos_100-1000mz.m | Operator | SCSIO |
| Sample Name | liqinglian_A3_pos | Instrument | maXis 255552.00029 |
| Comment | | | |

**Acquisition Parameter**

| | | | | | |
|---|---|---|---|---|---|
| Source Type | ESI | Ion Polarity | Positive | Set Nebulizer | 0.4 Bar |
| Focus | Active | Set Capillary | 4500 V | Set Dry Heater | 180 °C |
| Scan Begin | 100 m/z | Set End Plate Offset | -500 V | Set Dry Gas | 4.0 l/min |
| Scan End | 2000 m/z | Set Charging Voltage | 0 V | Set Divert Valve | Waste |
| | | Set Corona | 0 nA | Set APCI Heater | 0 °C |

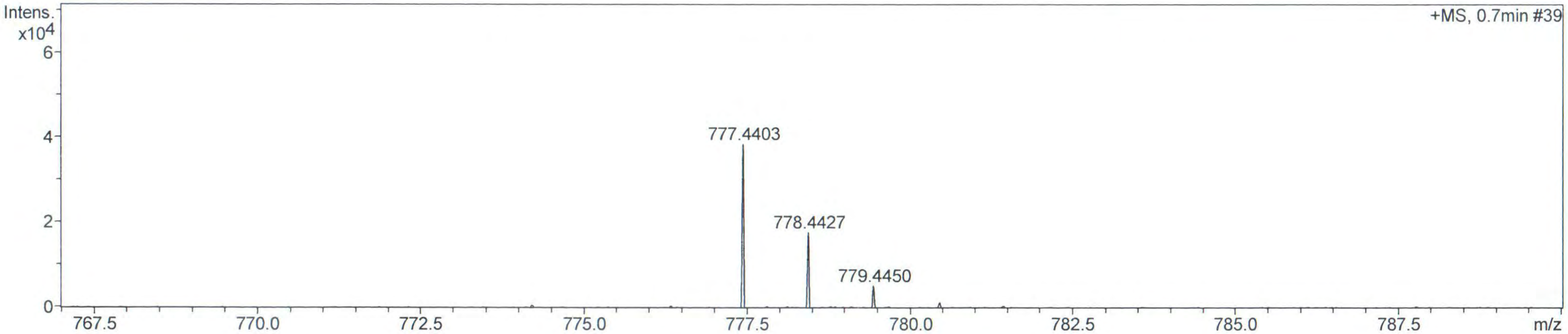


| Meas. m/z | # | Ion Formula | Score | m/z | err [ppm] | err [mDa] | mSigma | rdb | e⁻ Conf | N-Rule |
|---|---|---|---|---|---|---|---|---|---|---|
| 777.4403 | 1 | C39H57N10O7 | 100.00 | 777.4406 | 0.5 | 0.4 | 8.8 | 16.5 | even | ok |

liqinglian_A3_pos_54_01_9995.d
Bruker Compass DataAnalysis 4.1 printed: 2/8/2021 2:53:40 PM by: SCSIO Page 1 of 1

Figures S17. The 1H NMR spectrum of 12 in DMSO-$d_6$

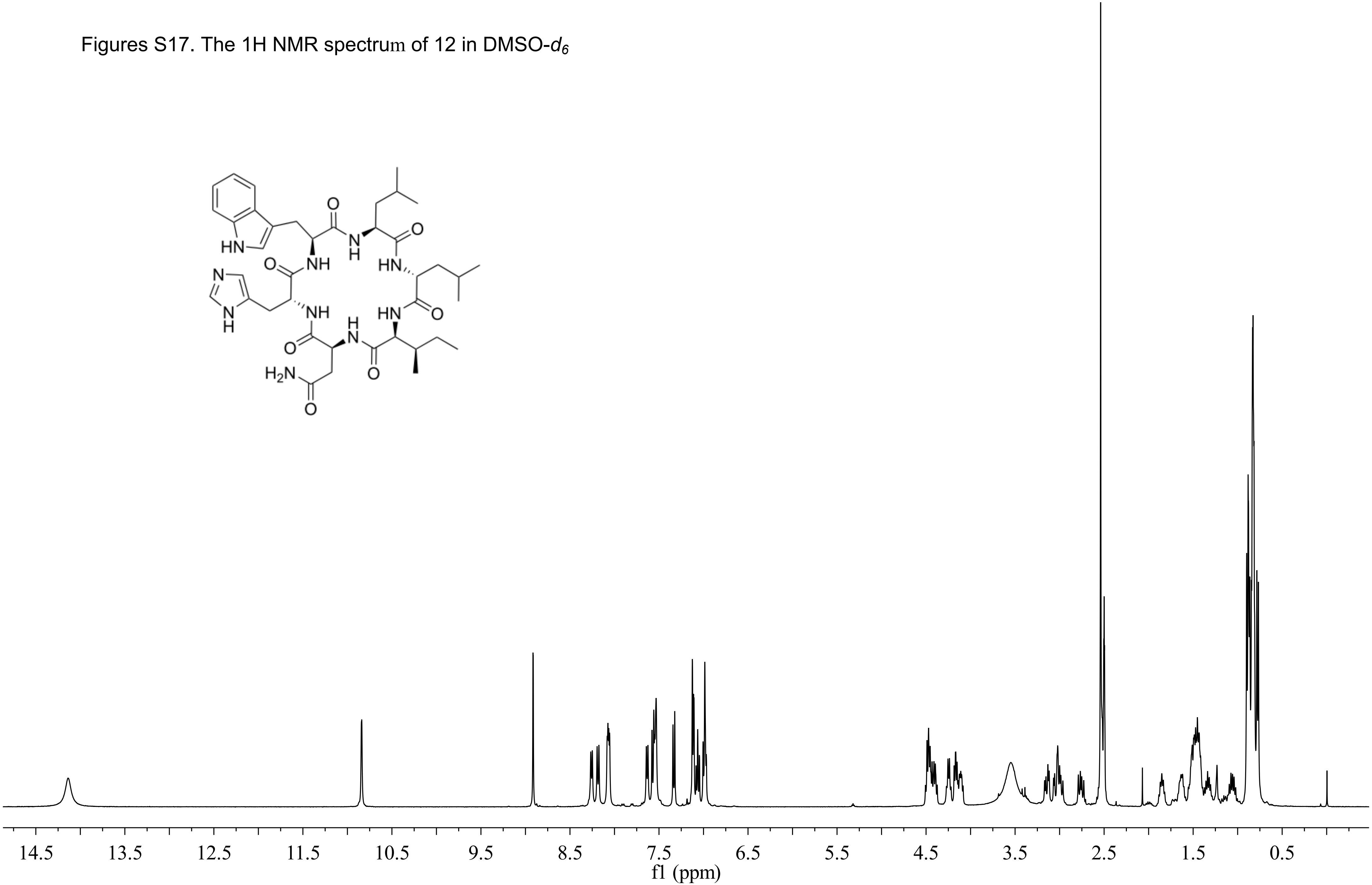

Figures S18. The 13C NMR spectrum of 12 in DMSO-$d_6$

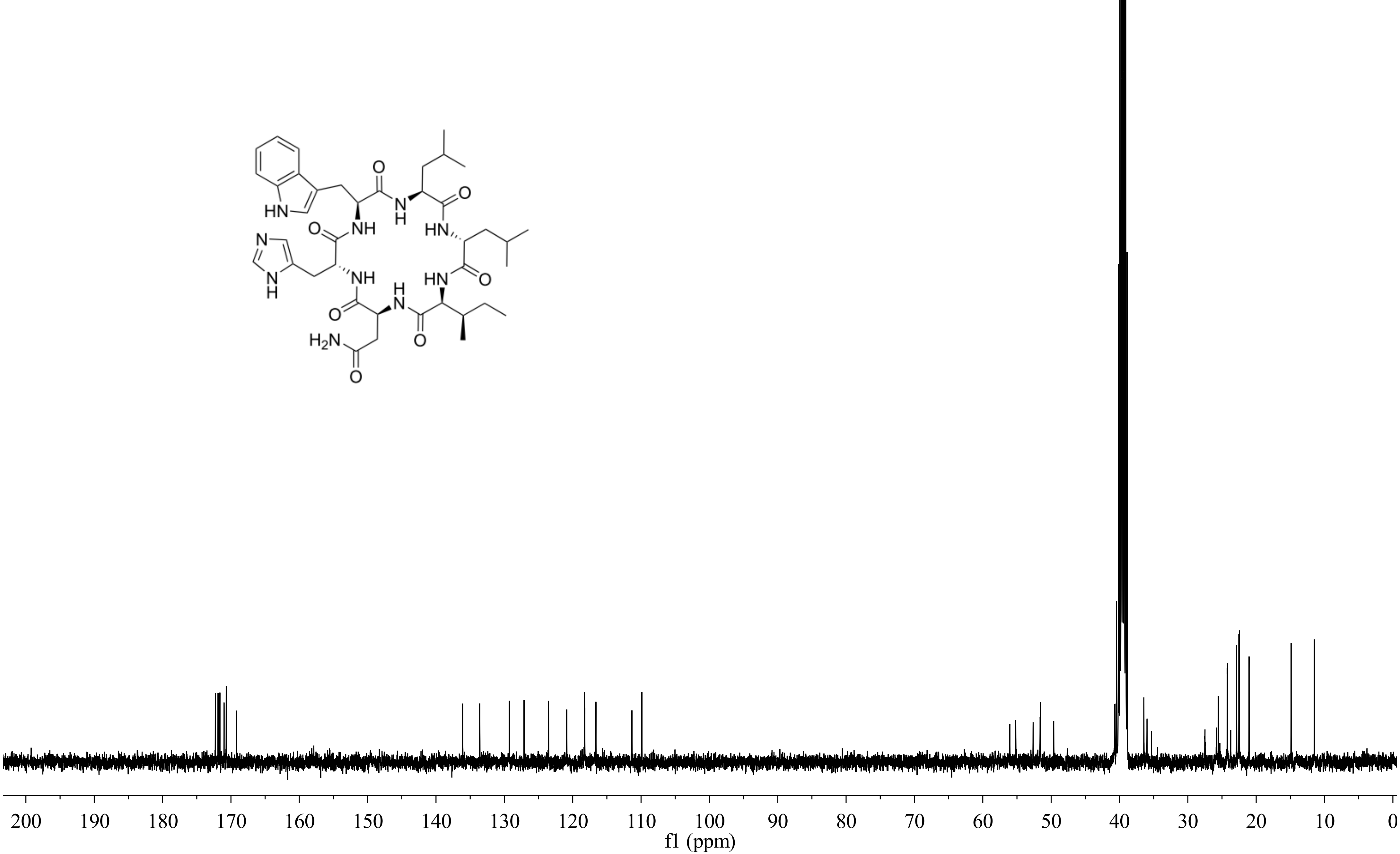

Figures S19. The HRESIMS spectrum of **13**

# Mass Spectrum SmartFormula Report

**Analysis Info**

| | | | |
|---|---|---|---|
| Analysis Name | D:\Data\MS\data\202102\liqinglian_A5_pos_55_01_9996.d | Acquisition Date | 2/8/2021 2:53:01 PM |
| Method | LC_Direct Infusion_pos_100-1000mz.m | Operator | SCSIO |
| Sample Name | liqinglian_A5_pos | Instrument | maXis 255552.00029 |
| Comment | | | |

**Acquisition Parameter**

| | | | | | |
|---|---|---|---|---|---|
| Source Type | ESI | Ion Polarity | Positive | Set Nebulizer | 0.4 Bar |
| Focus | Active | Set Capillary | 4500 V | Set Dry Heater | 180 °C |
| Scan Begin | 100 m/z | Set End Plate Offset | -500 V | Set Dry Gas | 4.0 l/min |
| Scan End | 2000 m/z | Set Charging Voltage | 0 V | Set Divert Valve | Waste |
| | | Set Corona | 0 nA | Set APCI Heater | 0 °C |

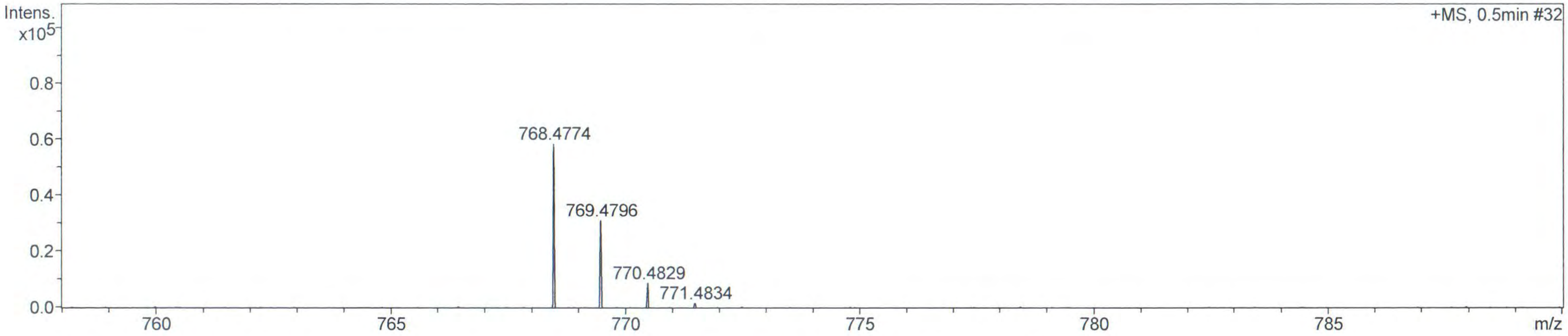


| Meas. m/z | # | Ion Formula | Score | m/z | err [ppm] | err [mDa] | mSigma | rdb | e⁻ Conf | N-Rule |
|---|---|---|---|---|---|---|---|---|---|---|
| 768.4774 | 1 | C39H62N9O7 | 100.00 | 768.4767 | 1.0 | 0.7 | 36.7 | 13.5 | even | ok |

liqinglian_A5_pos_55_01_9996.d
Bruker Compass DataAnalysis 4.1 printed: 2/8/2021 2:57:14 PM by: SCSIO Page 1 of 1

Figures S20. The 1H NMR spectrum of 13 in DMSO-$d_6$

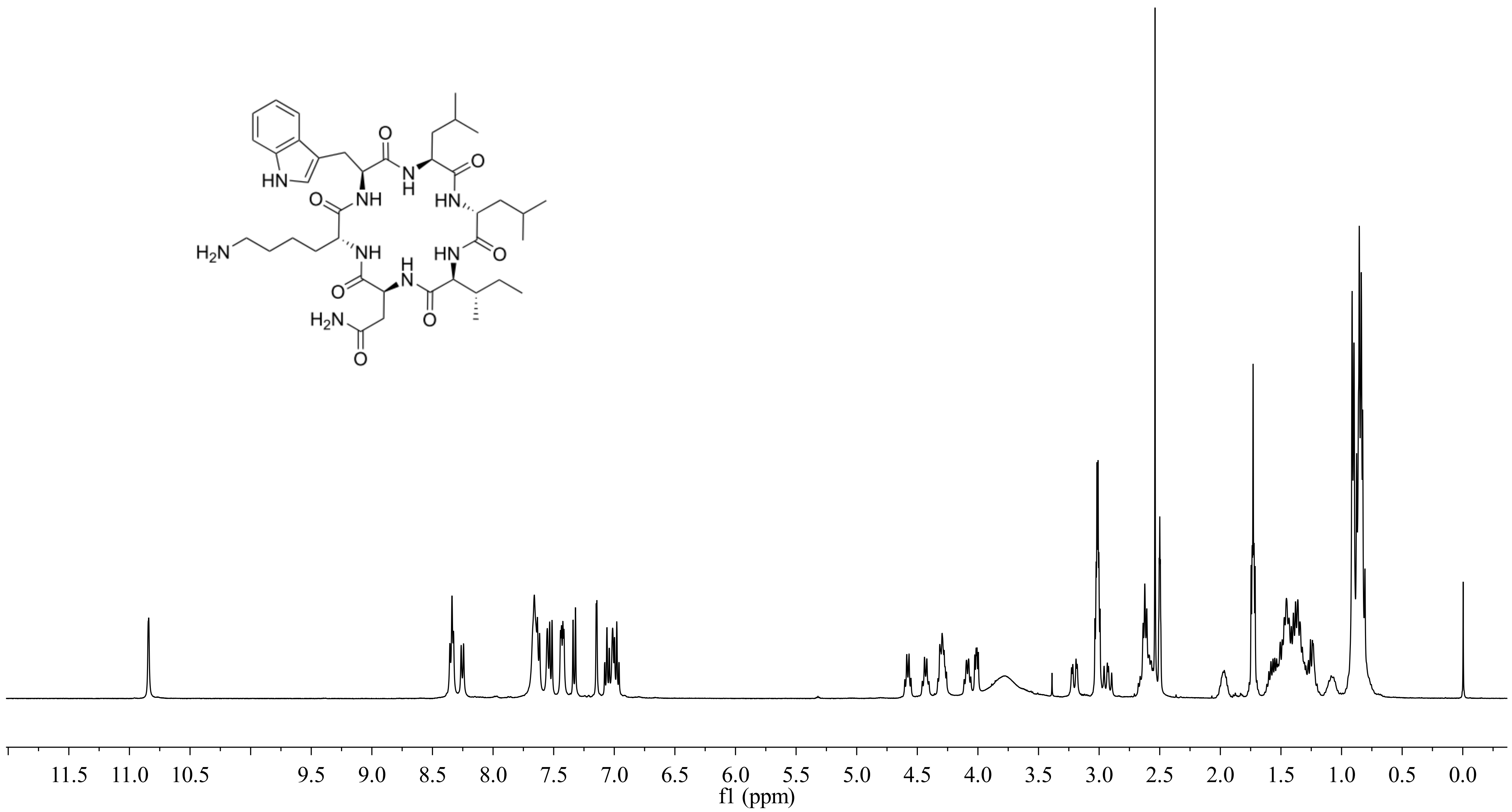

Figures S21. The 13C NMR spectrum of 13 in DMSO-$d_6$

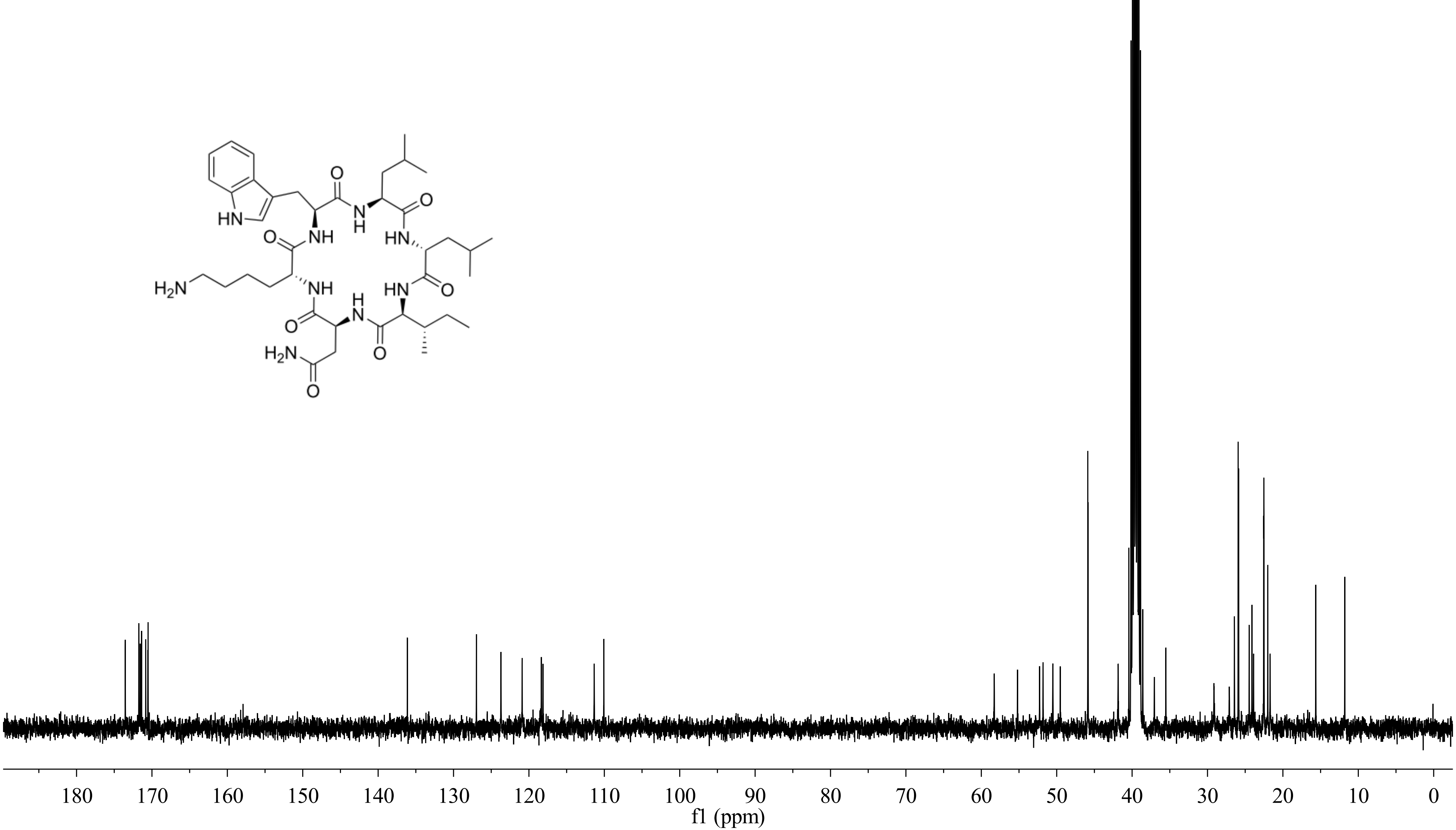

Figures S22. The HRESIMS spectrum of **14**

# Mass Spectrum SmartFormula Report

**Analysis Info**

| | | | | |
|---|---|---|---|---|
| Analysis Name | D:\Data\MS\data\202102\liqinglian_A6_pos_56_01_9997.d | Acquisition Date | 2/8/2021 2:56:29 PM | |
| Method | LC_Direct Infusion_pos_100-1000mz.m | Operator | SCSIO | |
| Sample Name | liqinglian_A6_pos | Instrument | maXis | 255552.00029 |
| Comment | | | | |

**Acquisition Parameter**

| | | | | | |
|---|---|---|---|---|---|
| Source Type | ESI | Ion Polarity | Positive | Set Nebulizer | 0.4 Bar |
| Focus | Active | Set Capillary | 4500 V | Set Dry Heater | 180 °C |
| Scan Begin | 100 m/z | Set End Plate Offset | -500 V | Set Dry Gas | 4.0 l/min |
| Scan End | 2000 m/z | Set Charging Voltage | 0 V | Set Divert Valve | Waste |
| | | Set Corona | 0 nA | Set APCI Heater | 0 °C |

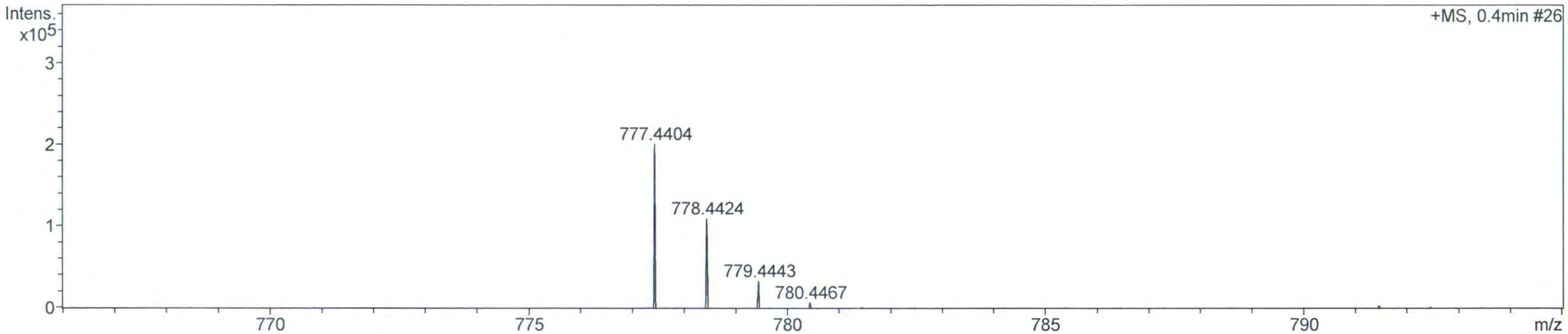


| Meas. m/z | # | Ion Formula | Score | m/z | err [ppm] | err [mDa] | mSigma | rdb | e⁻ Conf | N-Rule |
|---|---|---|---|---|---|---|---|---|---|---|
| 777.4404 | 1 | C39H57N10O7 | 100.00 | 777.4406 | 0.3 | 0.2 | 45.5 | 16.5 | even | ok |

liqinglian_A6_pos_56_01_9997.d

Bruker Compass DataAnalysis 4.1 printed: 2/8/2021 3:02:06 PM by: SCSIO Page 1 of 1

Figures S23. The 1H NMR spectrum of 14 in DMSO-$d_6$

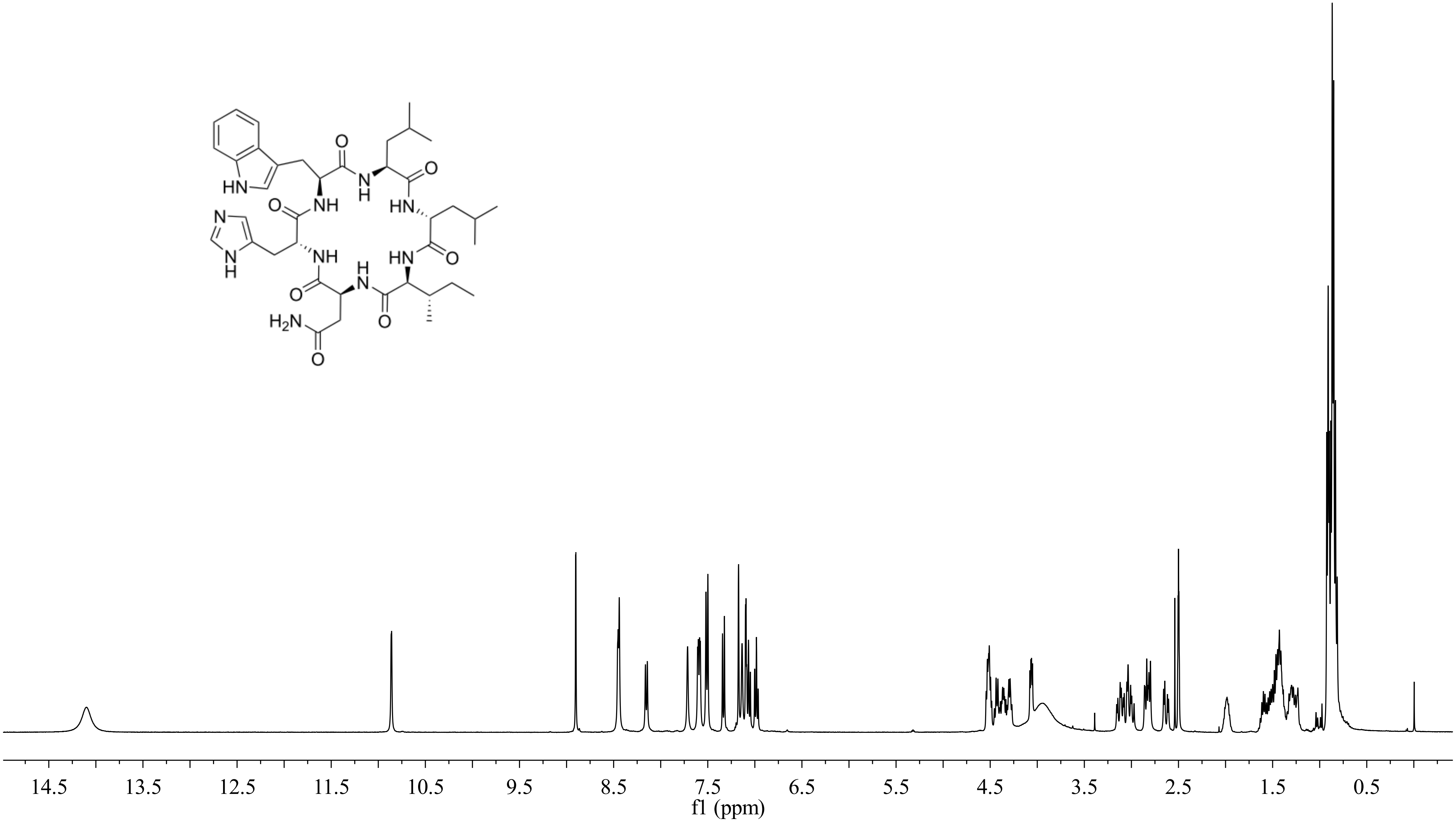

Figures S24. The 13C NMR spectrum of 14 in DMSO-$d_6$

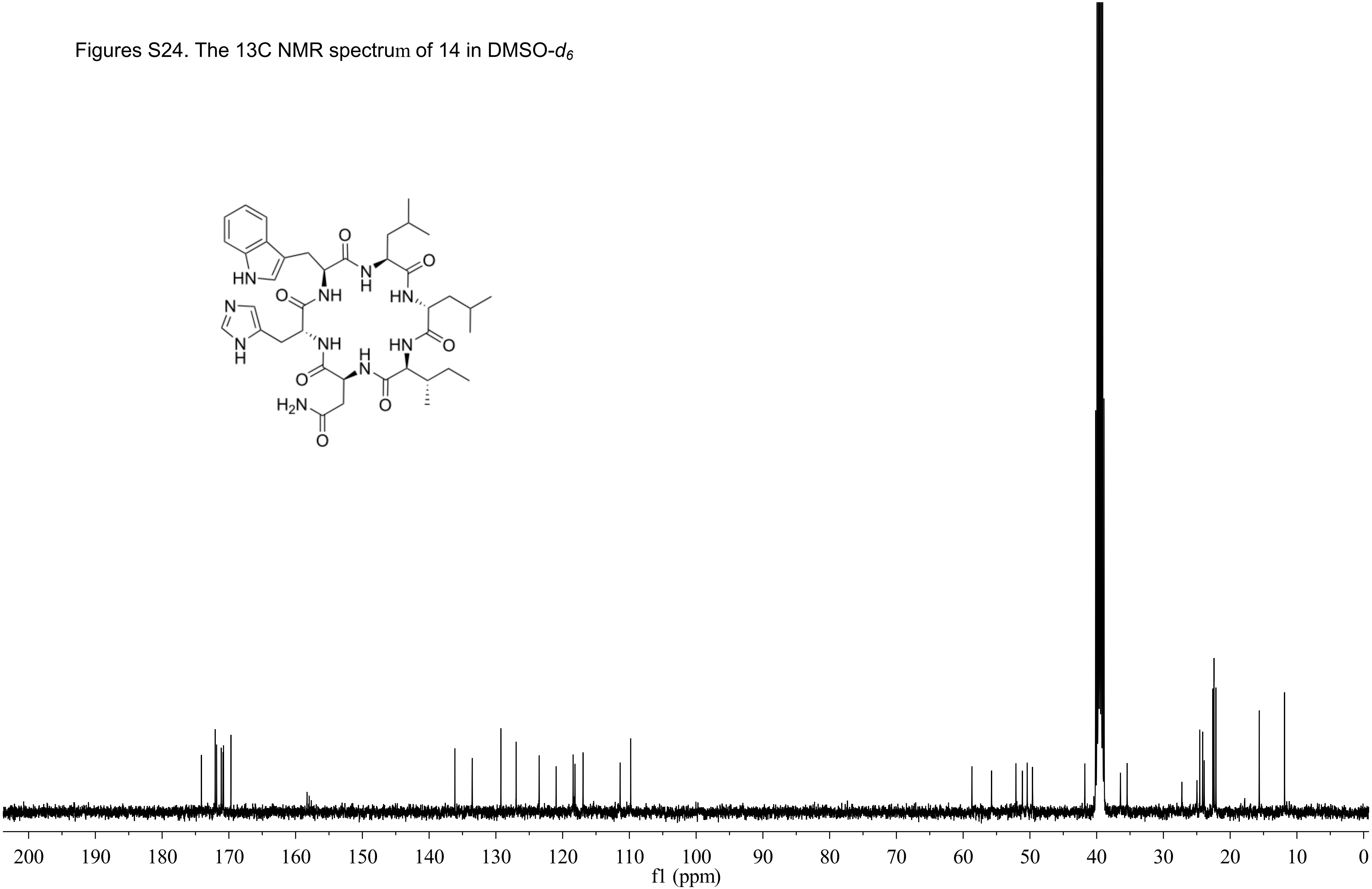

Figures S25. The HRESIMS spectrum of **15**

# Mass Spectrum SmartFormula Report

**Analysis Info**

| | | | |
|---|---|---|---|
| Analysis Name | D:\Data\MS\data\202102\liqinglian_A7_pos_57_01_9998.d | Acquisition Date | 2/8/2021 2:59:58 PM |
| Method | LC_Direct Infusion_pos_100-1000mz.m | Operator | SCSIO |
| Sample Name | liqinglian_A7_pos | Instrument | maXis 255552.00029 |
| Comment | | | |

**Acquisition Parameter**

| | | | | | |
|---|---|---|---|---|---|
| Source Type | ESI | Ion Polarity | Positive | Set Nebulizer | 0.4 Bar |
| Focus | Active | Set Capillary | 4500 V | Set Dry Heater | 180 °C |
| Scan Begin | 100 m/z | Set End Plate Offset | -500 V | Set Dry Gas | 4.0 l/min |
| Scan End | 2000 m/z | Set Charging Voltage | 0 V | Set Divert Valve | Waste |
| | | Set Corona | 0 nA | Set APCI Heater | 0 °C |

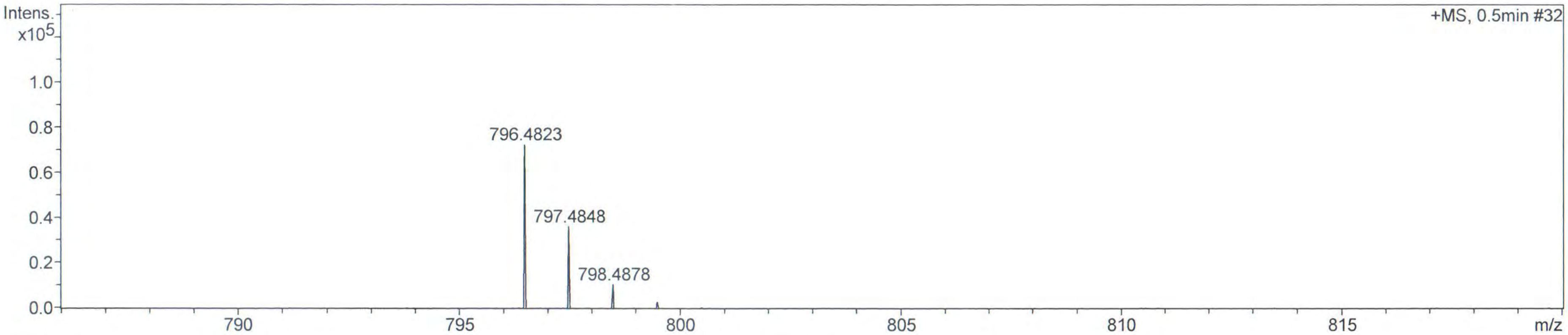


| Meas. m/z | # | Ion Formula | Score | m/z | err [ppm] | err [mDa] | mSigma | rdb | e⁻ Conf | N-Rule |
|---|---|---|---|---|---|---|---|---|---|---|
| 796.4823 | 1 | C39H62N11O7 | 100.00 | 796.4828 | -0.6 | -0.5 | 19.6 | 14.5 | even | ok |

liqinglian_A7_pos_57_01_9998.d

Bruker Compass DataAnalysis 4.1 printed: 2/8/2021 3:06:03 PM by: SCSIO Page 1 of 1

Figures S26. The 1H NMR spectrum of 15 in DMSO-$d_6$

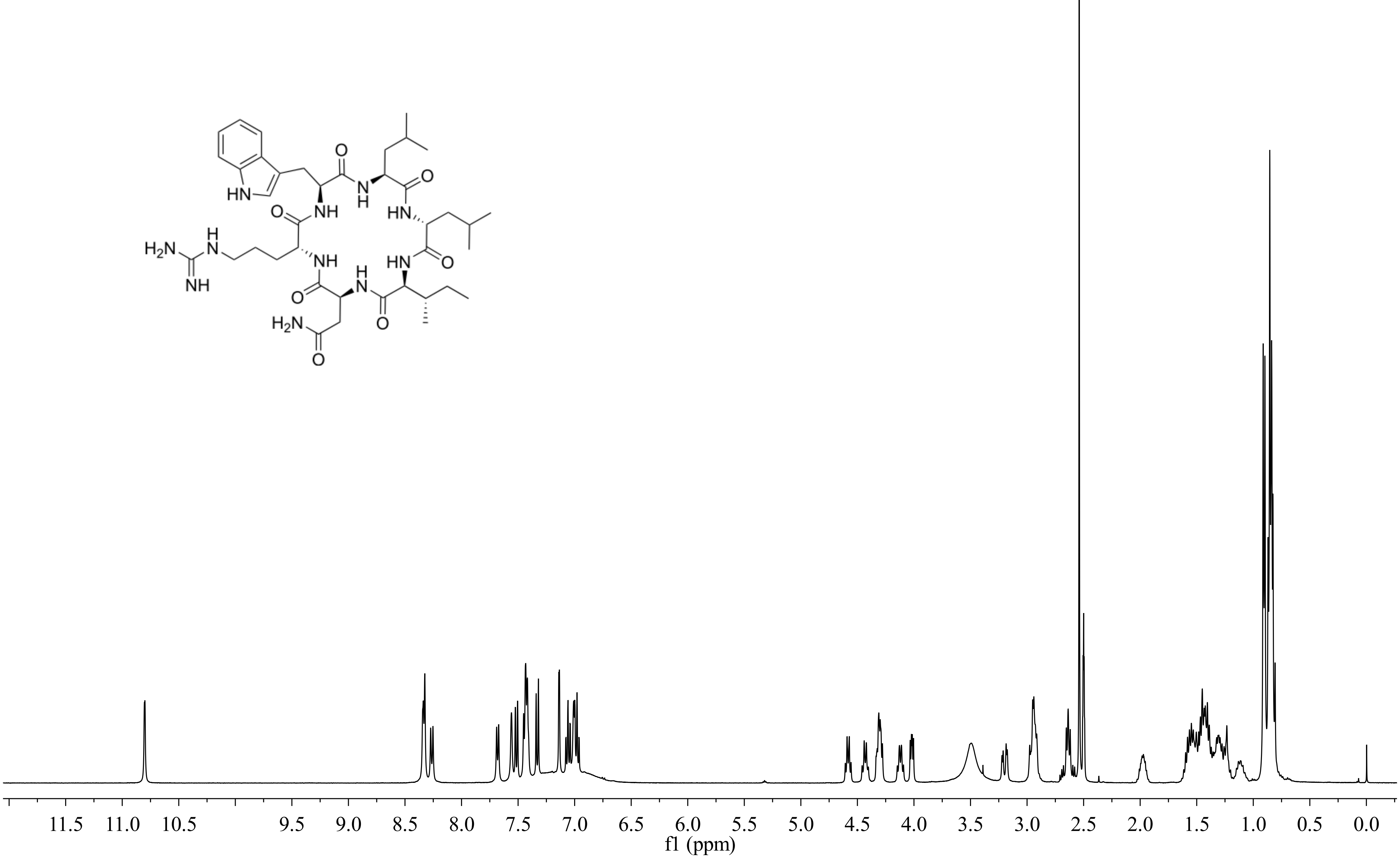

Figures S27. The 13C NMR spectrum of 15 in DMSO-$d_6$

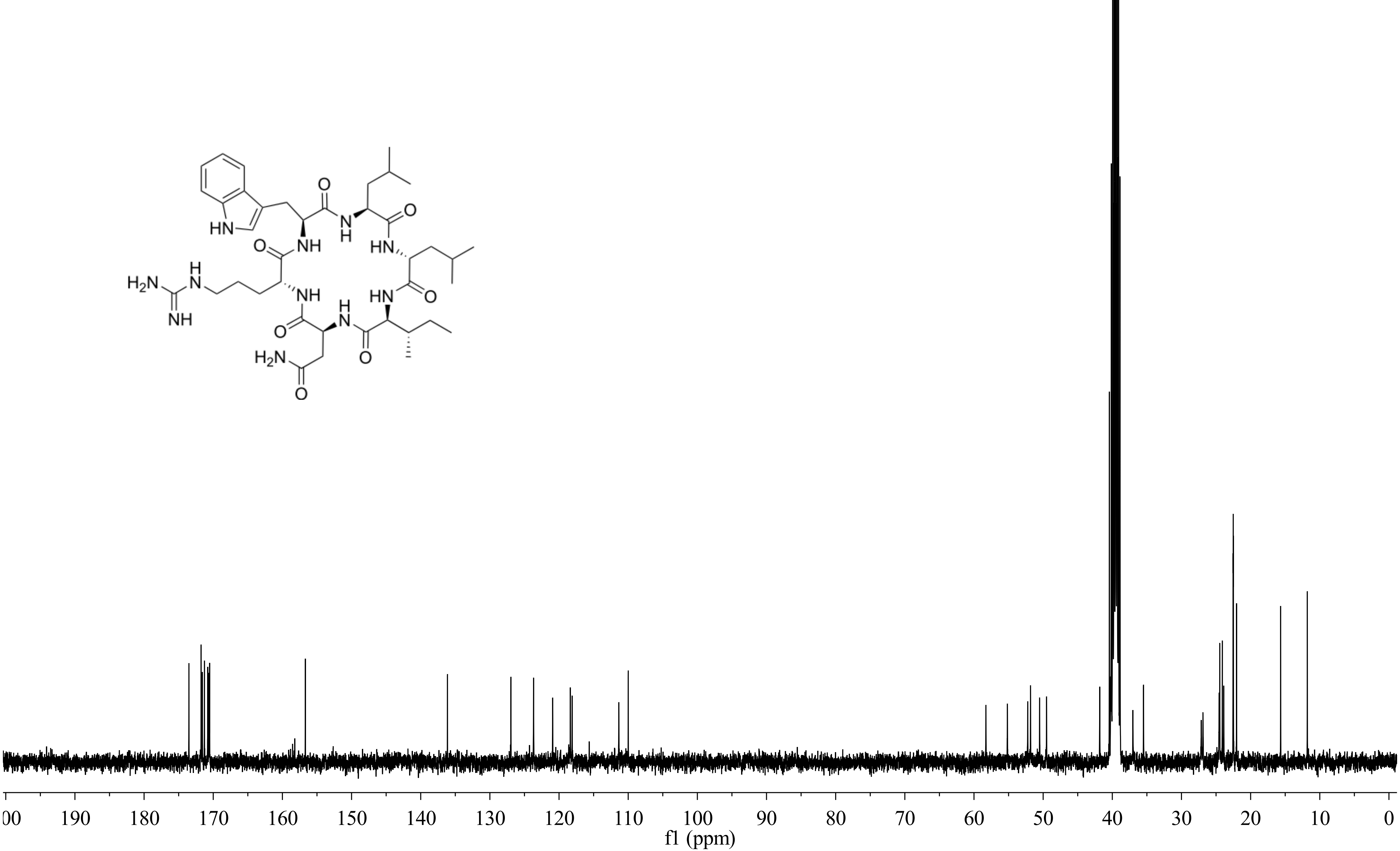

Figures S28. The HRESIMS spectrum of **16**

# Mass Spectrum SmartFormula Report

**Analysis Info**

| | | | |
|---|---|---|---|
| Analysis Name | D:\Data\MS\data\202102\liqinglian_A8_pos_58_01_9999.d | Acquisition Date | 2/8/2021 3:03:27 PM |
| Method | LC_Direct Infusion_pos_100-1000mz.m | Operator | SCSIO |
| Sample Name | liqinglian_A8_pos | Instrument | maXis 255552.00029 |
| Comment | | | |

**Acquisition Parameter**

| | | | | | |
|---|---|---|---|---|---|
| Source Type | ESI | Ion Polarity | Positive | Set Nebulizer | 0.4 Bar |
| Focus | Active | Set Capillary | 4500 V | Set Dry Heater | 180 °C |
| Scan Begin | 100 m/z | Set End Plate Offset | -500 V | Set Dry Gas | 4.0 l/min |
| Scan End | 2000 m/z | Set Charging Voltage | 0 V | Set Divert Valve | Waste |
| | | Set Corona | 0 nA | Set APCI Heater | 0 °C |

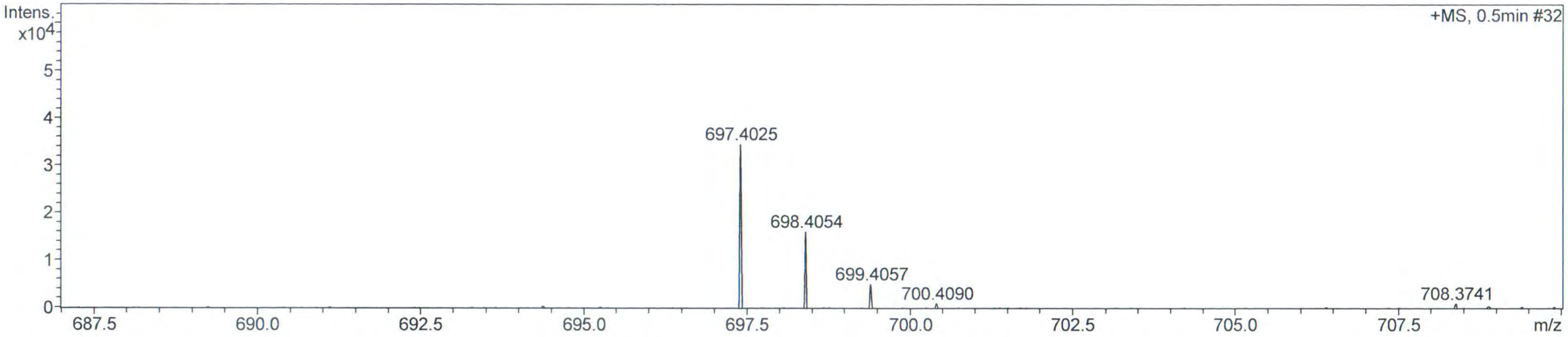


| Meas. m/z | # | Ion Formula | Score | m/z | err [ppm] | err [mDa] | mSigma | rdb | e⁻ Conf | N-Rule |
|---|---|---|---|---|---|---|---|---|---|---|
| 697.4025 | 1 | C35H53N8O7 | 100.00 | 697.4032 | -1.0 | -0.7 | 35.2 | 13.5 | even | ok |

Figures S29. The 1H NMR spectrum of 16 in DMSO-$d_6$

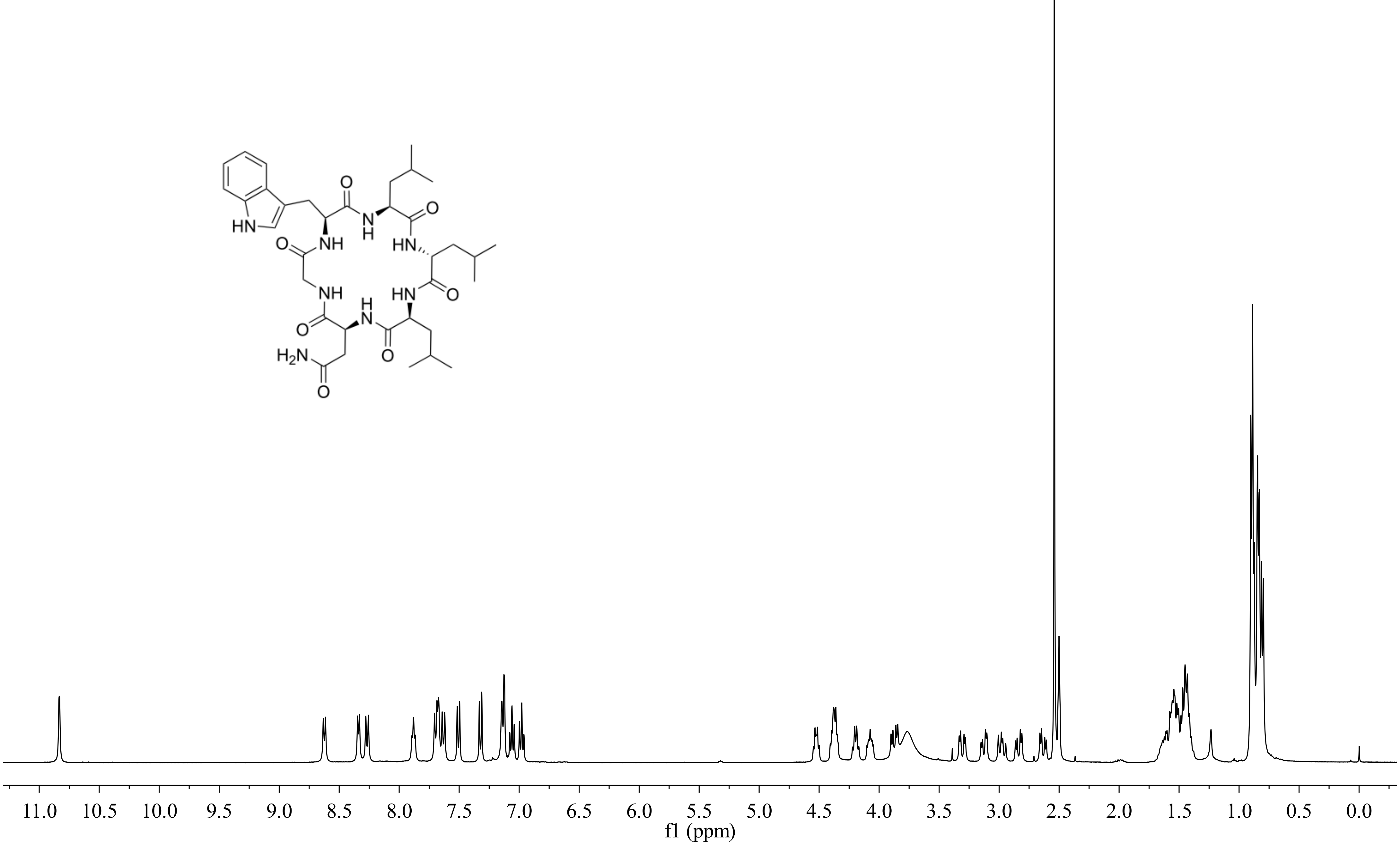

Figures S30. The 13C NMR spectrum of 16 in DMSO-$d_6$

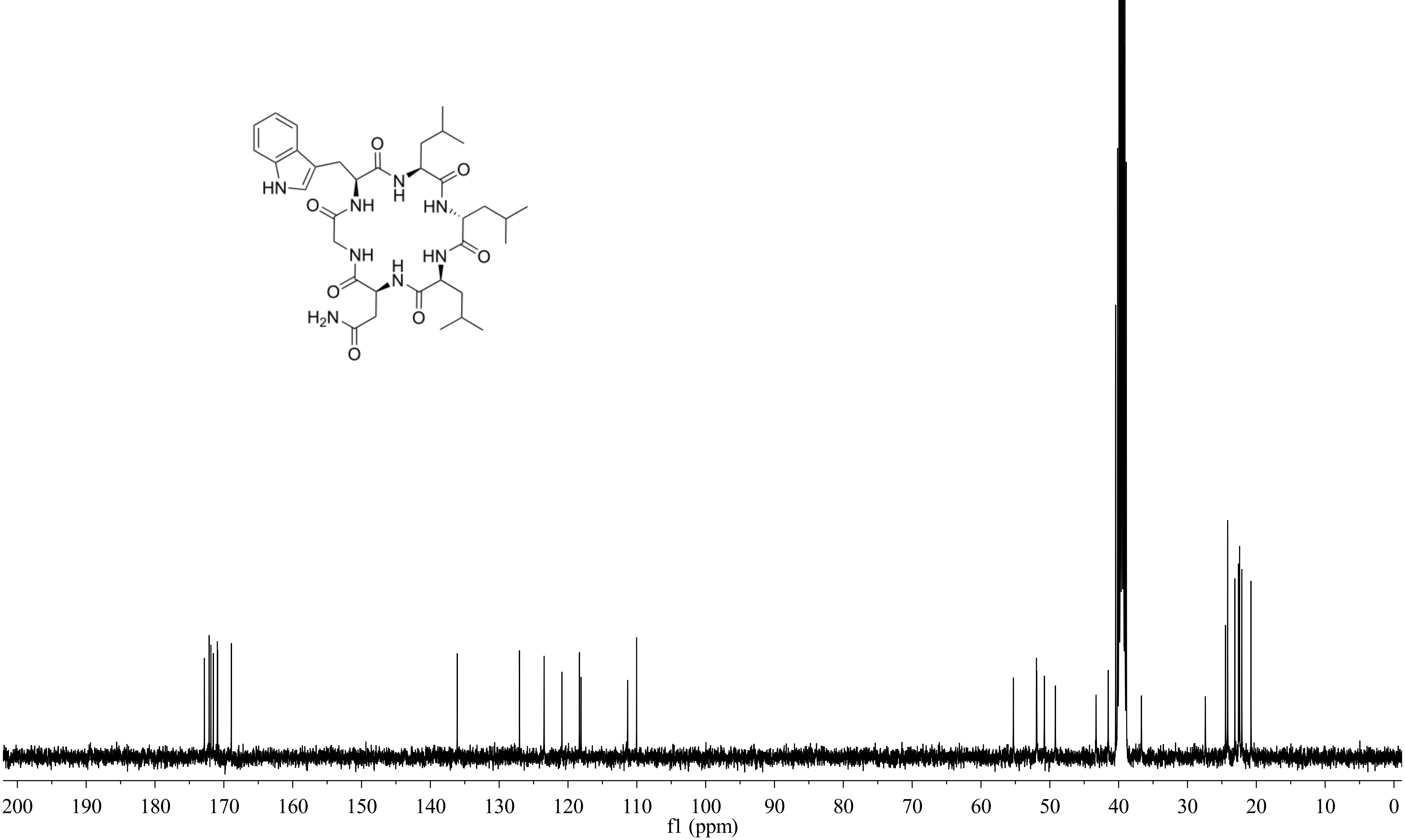

Figures S31. The HRESIMS spectrum of **17**

# Mass Spectrum SmartFormula Report

**Analysis Info**

| | | | |
|---|---|---|---|
| Analysis Name | D:\Data\MS\data\202102\liqinglian_A9_pos_59_01_10000.d | Acquisition Date | 2/8/2021 3:06:57 PM |
| Method | LC_Direct Infusion_pos_100-1000mz.m | Operator | SCSIO |
| Sample Name | liqinglian_A9_pos | Instrument | maXis 255552.00029 |
| Comment | | | |

**Acquisition Parameter**

| | | | | | |
|---|---|---|---|---|---|
| Source Type | ESI | Ion Polarity | Positive | Set Nebulizer | 0.4 Bar |
| Focus | Active | Set Capillary | 4500 V | Set Dry Heater | 180 °C |
| Scan Begin | 100 m/z | Set End Plate Offset | -500 V | Set Dry Gas | 4.0 l/min |
| Scan End | 2000 m/z | Set Charging Voltage | 0 V | Set Divert Valve | Waste |
| | | Set Corona | 0 nA | Set APCI Heater | 0 °C |

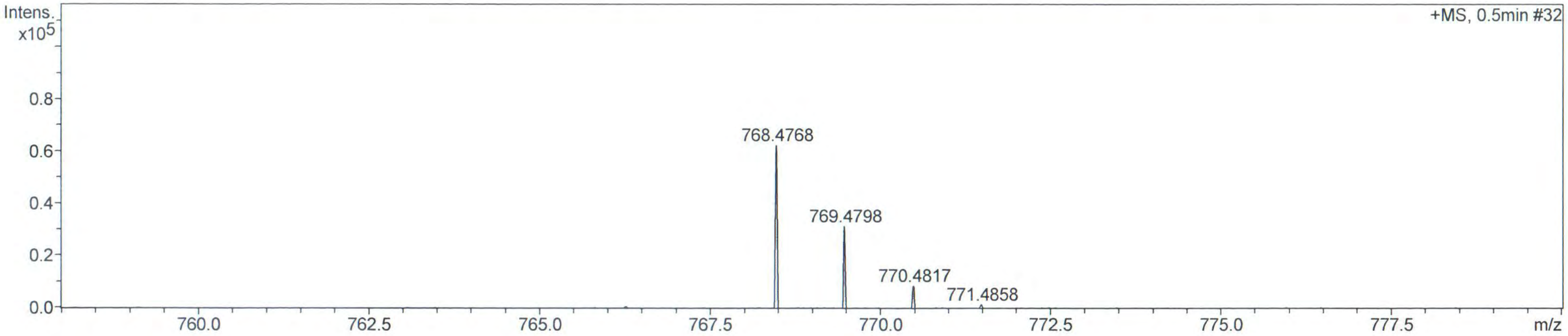


| Meas. m/z | # | Ion Formula | Score | m/z | err [ppm] | err [mDa] | mSigma | rdb | e⁻ Conf | N-Rule |
|---|---|---|---|---|---|---|---|---|---|---|
| 768.4768 | 1 | C39H62N9O7 | 100.00 | 768.4767 | 0.1 | 0.1 | 20.2 | 13.5 | even | ok |

liqinglian_A9_pos_59_01_10000.d

Bruker Compass DataAnalysis 4.1 printed: 2/8/2021 3:10:15 PM by: SCSIO Page 1 of 1

Figures S32. The 1H NMR spectrum of 17 in DMSO-$d_6$

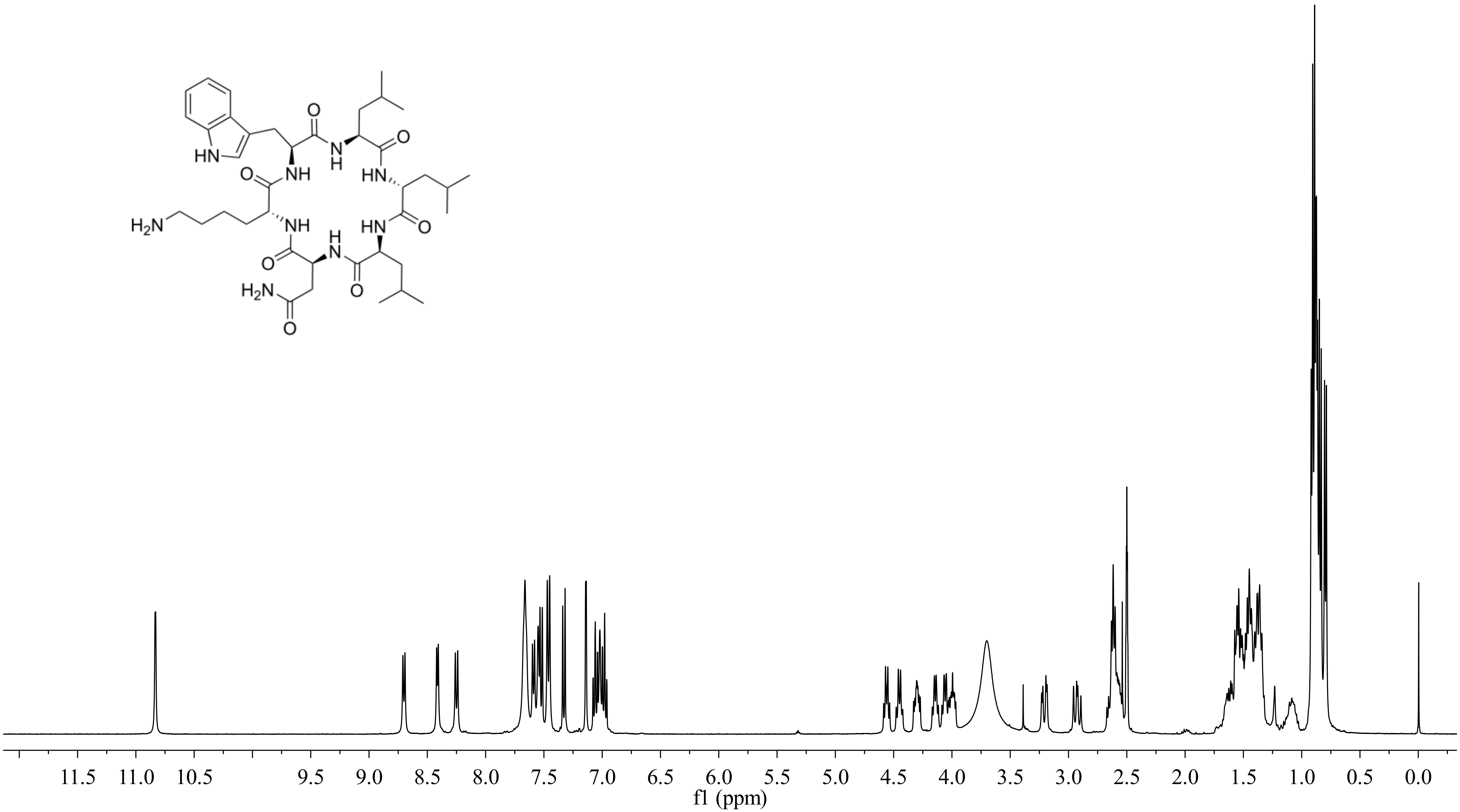

Figures S33. The 13C NMR spectrum of 17 in DMSO-$d_6$

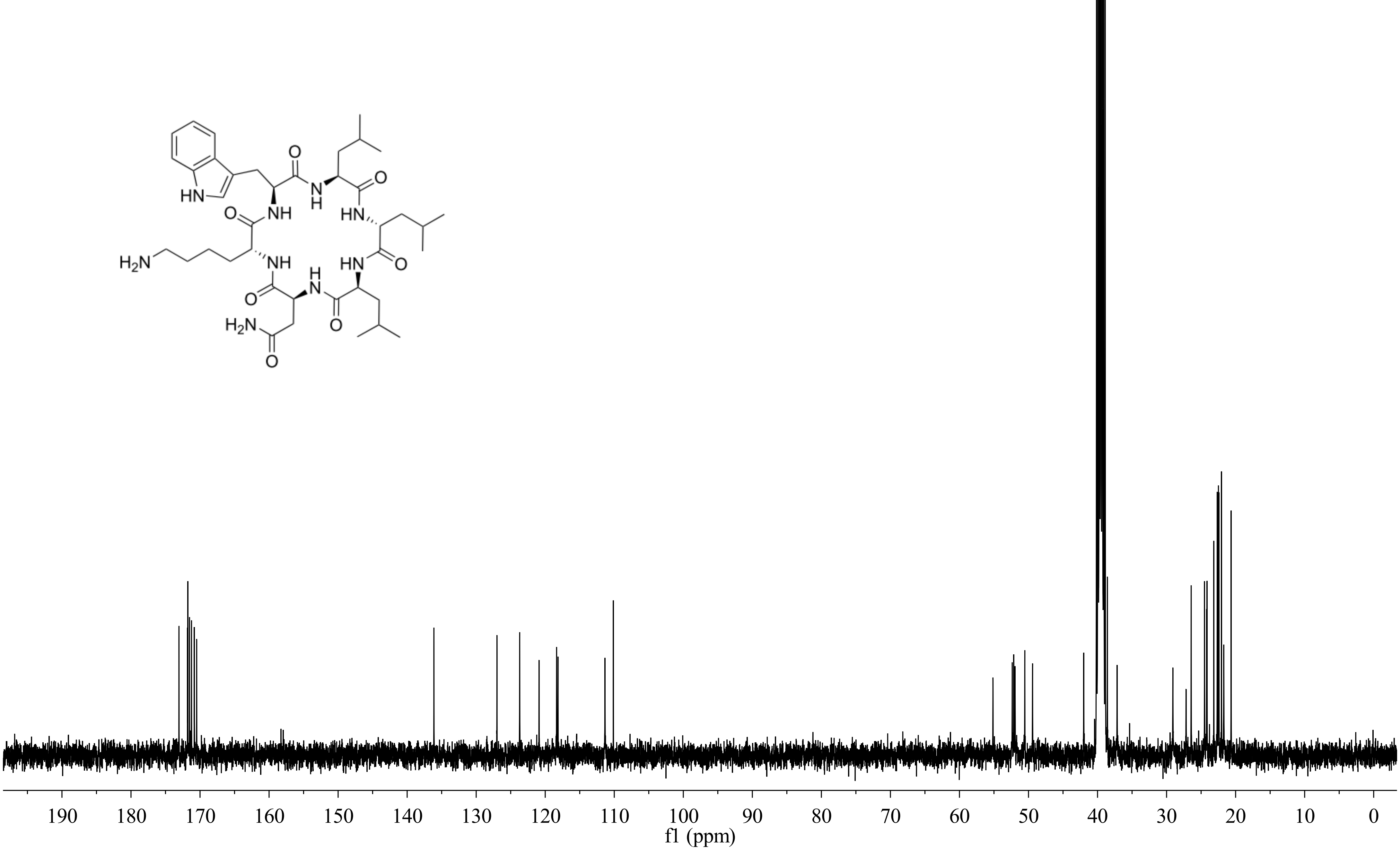

Figures S34. The HRESIMS spectrum of **18**

# Mass Spectrum SmartFormula Report

**Analysis Info**

| | | | |
|---|---|---|---|
| Analysis Name | D:\Data\MS\data\202102\liqinglian_A10_pos_60_01_10001.d | Acquisition Date | 2/8/2021 3:10:24 PM |
| Method | LC_Direct Infusion_pos_100-1000mz.m | Operator | SCSIO |
| Sample Name | liqinglian_A10_pos | Instrument | maXis 255552.00029 |
| Comment | | | |

**Acquisition Parameter**

| | | | | | |
|---|---|---|---|---|---|
| Source Type | ESI | Ion Polarity | Positive | Set Nebulizer | 0.4 Bar |
| Focus | Active | Set Capillary | 4500 V | Set Dry Heater | 180 °C |
| Scan Begin | 100 m/z | Set End Plate Offset | -500 V | Set Dry Gas | 4.0 l/min |
| Scan End | 2000 m/z | Set Charging Voltage | 0 V | Set Divert Valve | Waste |
| | | Set Corona | 0 nA | Set APCI Heater | 0 °C |

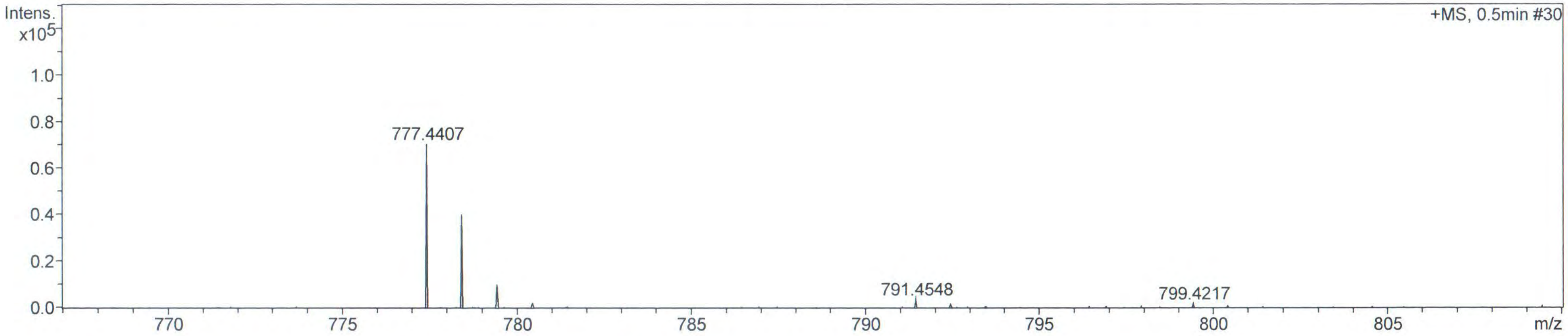


| Meas. m/z | # | Ion Formula | Score | m/z | err [ppm] | err [mDa] | mSigma | rdb | e¯ Conf | N-Rule |
|---|---|---|---|---|---|---|---|---|---|---|
| 777.4407 | 1 | C39H57N10O7 | 100.00 | 777.4406 | -0.1 | -0.1 | 53.4 | 16.5 | even | ok |
| 799.4217 | 1 | C39H56N10NaO7 | 100.00 | 799.4226 | 1.1 | 0.8 | 40.7 | 16.5 | even | ok |

liqinglian_A10_pos_60_01_10001.d
Bruker Compass DataAnalysis 4.1 printed: 2/8/2021 3:15:05 PM by: SCSIO Page 1 of 1

Figures S35. The 1H NMR spectrum of 18 in DMSO-$d_6$

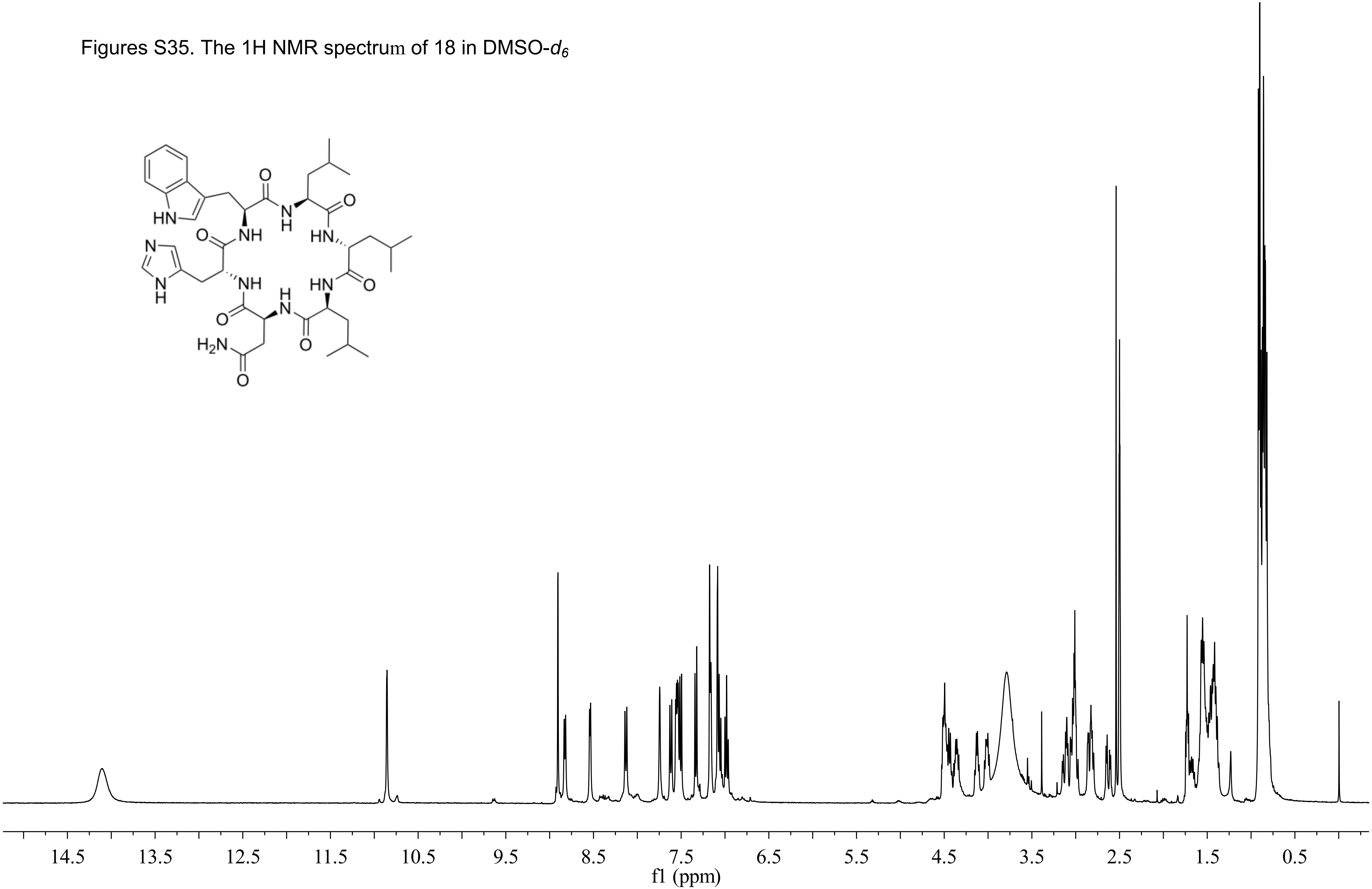

Figures S36. The 13C NMR spectrum of 18 in DMSO-$d_6$

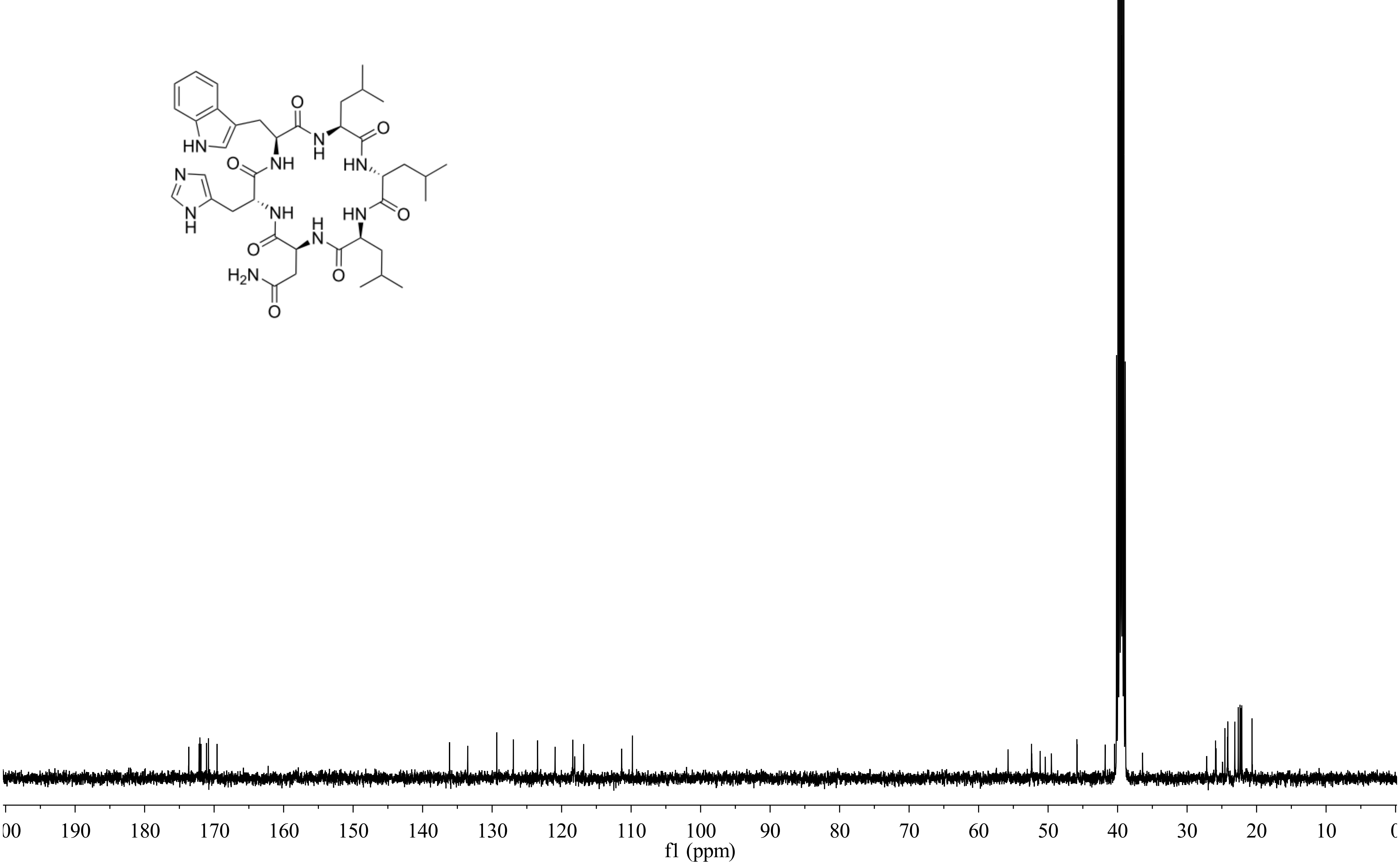

Figures S37. The HRESIMS spectrum of **19**

# Mass Spectrum SmartFormula Report

**Analysis Info**

| | | | |
|---|---|---|---|
| Analysis Name | D:\Data\MS\data\202102\liqinglian_A11_pos_45_01_10002.d | Acquisition Date | 2/8/2021 3:13:53 PM |
| Method | LC_Direct Infusion_pos_100-1000mz.m | Operator | SCSIO |
| Sample Name | liqinglian_A11_pos | Instrument | maXis 255552.00029 |
| Comment | | | |

**Acquisition Parameter**

| | | | | | |
|---|---|---|---|---|---|
| Source Type | ESI | Ion Polarity | Positive | Set Nebulizer | 0.4 Bar |
| Focus | Active | Set Capillary | 4500 V | Set Dry Heater | 180 °C |
| Scan Begin | 100 m/z | Set End Plate Offset | -500 V | Set Dry Gas | 4.0 l/min |
| Scan End | 2000 m/z | Set Charging Voltage | 0 V | Set Divert Valve | Waste |
| | | Set Corona | 0 nA | Set APCI Heater | 0 °C |

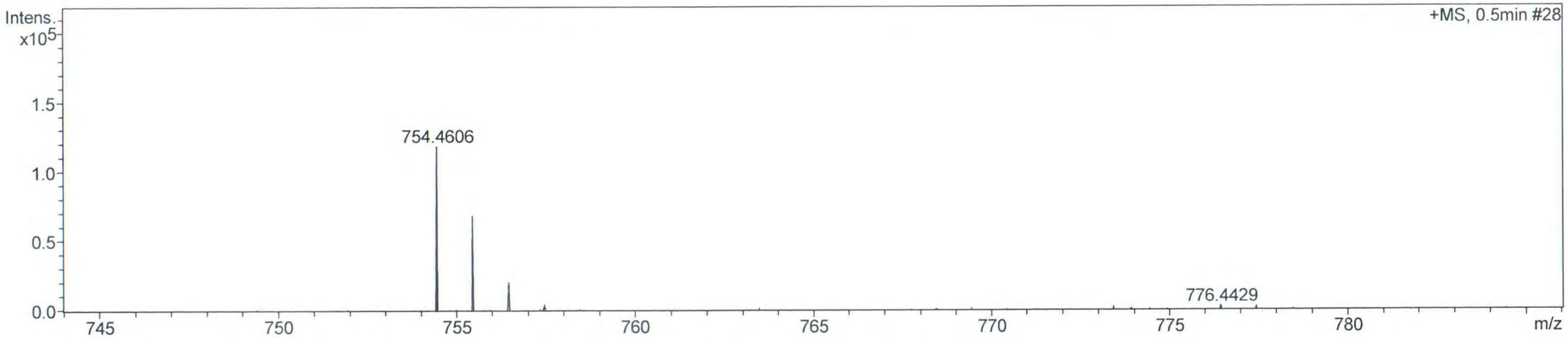


| Meas. m/z | # | Ion Formula | Score | m/z | err [ppm] | err [mDa] | mSigma | rdb | e⁻ Conf | N-Rule |
|---|---|---|---|---|---|---|---|---|---|---|
| 754.4606 | 1 | C38H60N9O7 | 100.00 | 754.4610 | -0.6 | -0.4 | 69.8 | 13.5 | even | ok |
| 776.4429 | 1 | C38H59N9NaO7 | 100.00 | 776.4430 | 0.0 | 0.0 | 156.2 | 13.5 | even | ok |

liqinglian_A11_pos_45_01_10002.d

Bruker Compass DataAnalysis 4.1 printed: 2/8/2021 3:17:29 PM by: SCSIO Page 1 of 1

Figures S38. The 1H NMR spectrum of 19 in DMSO-$d_6$

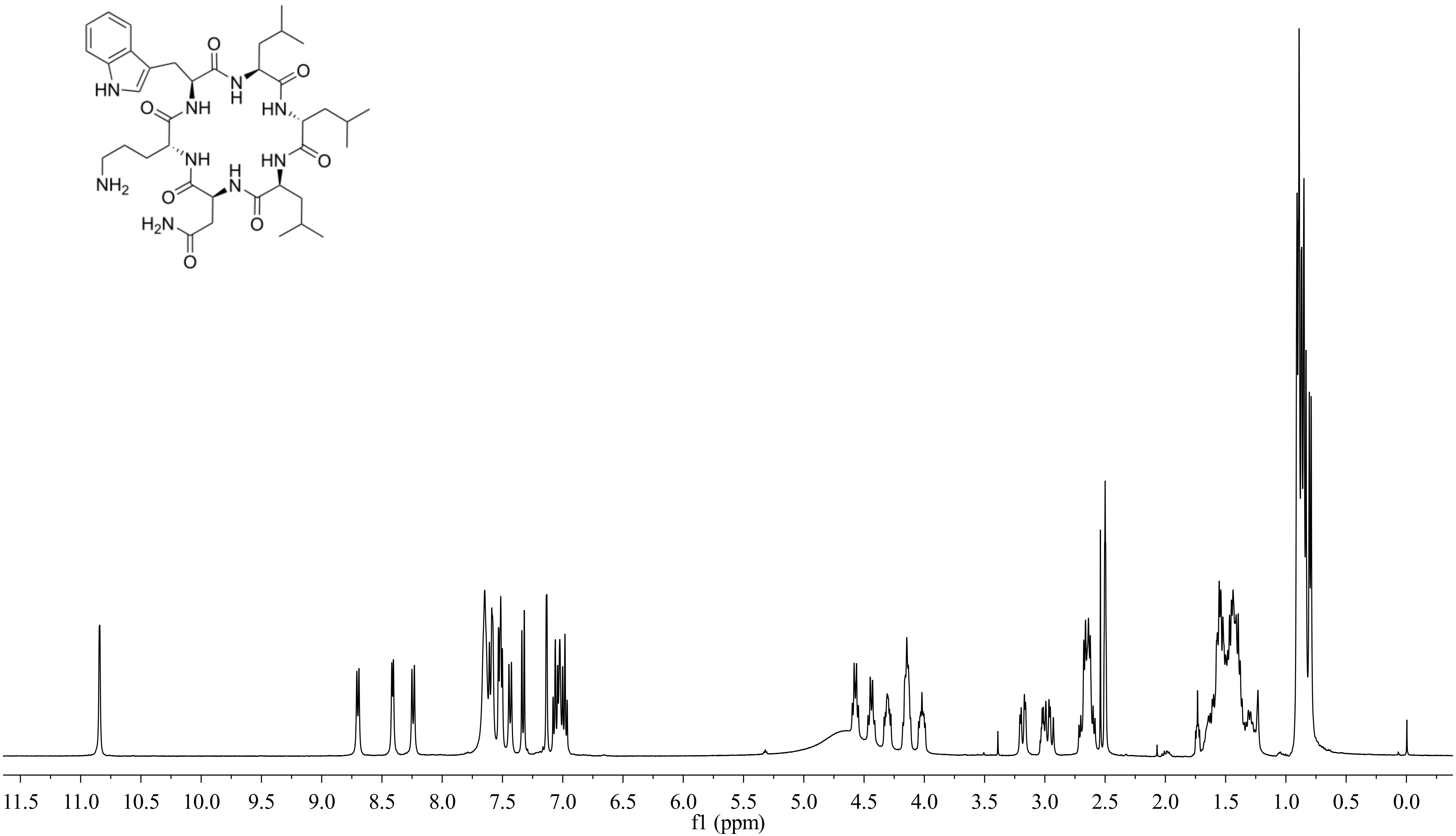

Figures S39. The 13C NMR spectrum of 19 in DMSO-$d_6$

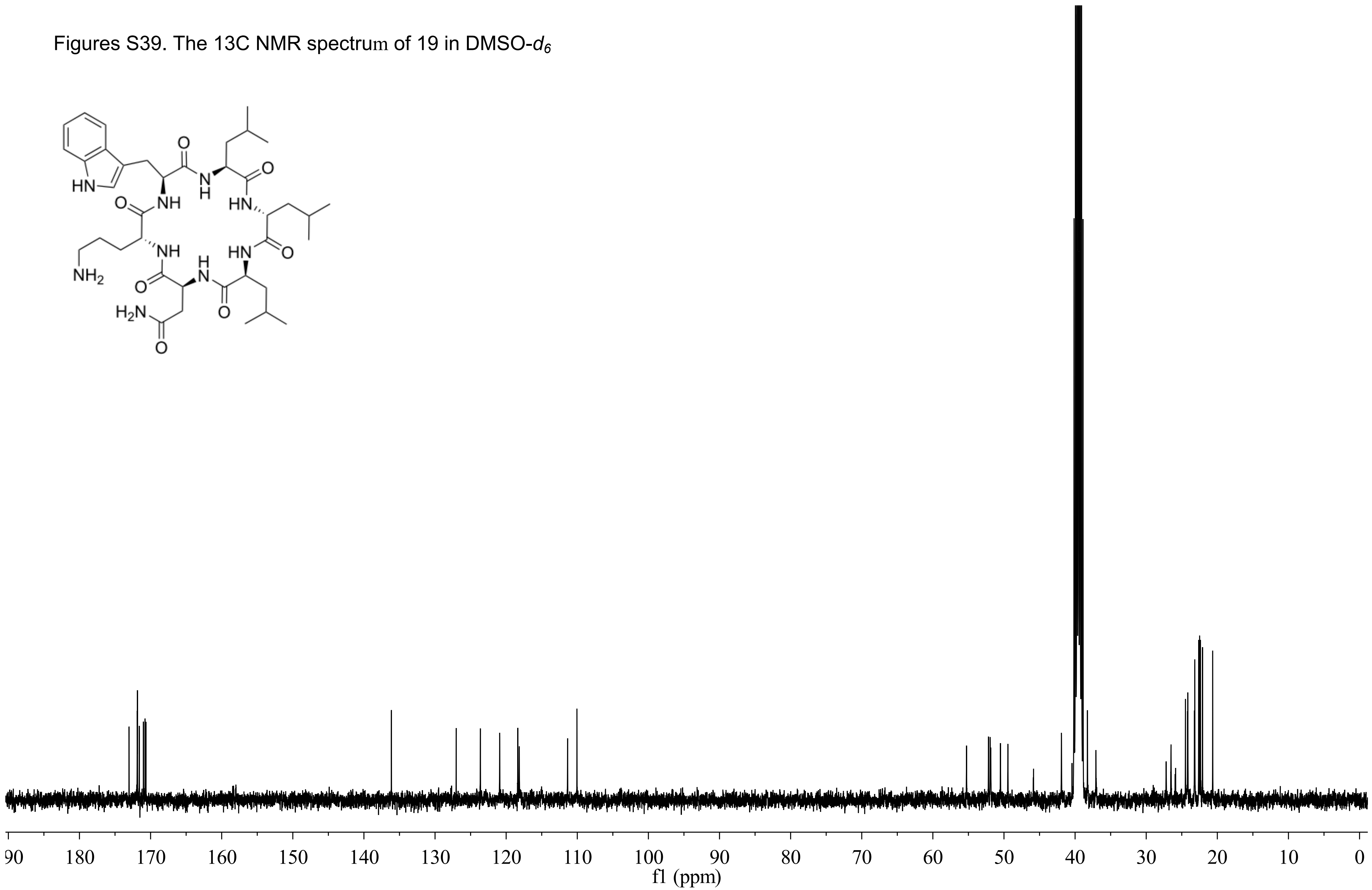

Supplement: Supplementary file 1 [file marinedrugs-19-00303-s001.zip › marinedrugs-1234633-supplementary.pdf]
